# Supplementary material for: Multifaceted antibiotic stewardship intervention using a participatory-action-research approach to improve antibiotic prescribing for urinary tract infections in frail elderly (ImpresU): study protocol for a European qualitative study followed by a pragmatic cluster randomised controlled trial
Source: BMJ Open. 2021 Oct 7;11(10):e052552. doi: 10.1136/bmjopen-2021-052552 (PMC8499316; doi:10.1136/bmjopen-2021-052552)

SUPPLEMENTARY DATA

Data supplement 1: Setting of the participating clusters in the cluster RCT

Poland

Participating clusters consist of nursing homes with patients registered to a particular GP primary care centre. Nursing homes in Poland provide living, care, support and educational services to people who require 24-hour care due to their age, illness or disability. Nursing homes may be conducted by local government units, churches, or other associations.

- Nursing care is provided 24-hours a day.
- Patients are registered to a particular GP in a primary care centre.
- Medical services are provided on the general principles of the National Health Fund. Patients can visit their GP in the centre or the GP comes to the nursing home on regular basis and on demand.
- During out-of hours, the regular GP/GP-practice is not be available. Instead, out-of hours service doctors are responsible or an ambulance is called in urgent cases.

The Netherlands

Participating clusters consist of residential care homes or home care organisation and their attending GP practices. This used to be a well-defined GP-attended setting; however, due to recent policy changes the setting is now quite heterogeneous. It does not include nursing homes; specialized elderly care physicians provide medical care in nursing homes.

- Patients receive varying degrees of ADL care, often provided by nurse-assistants with lower educational levels compared to the nursing home setting. Often, nurses are available (on-call). Patients may live in residential care homes or apartment complexes next to it, small-scale living facilities for dementia care, or have “regular” homes with access to home care.
- Medical care is provided by the GP. Often, more than one GP practice is connected to the nursing teams, as patients choose their own GP and their own nursing care organisation. In some residential care homes, the GP visits on a regular basis, for others, the GP is available only on demand.
- During out-of hours, the out-of-hour GP service is available instead of the regular GP.

Norway

Participating clusters consist of nursing homes with nursing home doctors providing medical care. Nursing homes are organised by municipalities, and are reserved for the most vulnerable older persons; those who need 24 hours surveillance and/or are severely dependent in ADL.

- 24-hour care is available at the nursing home from nurses and nurse assistants.
- Medical care is provided by nursing home doctors, with various medical backgrounds, e.g. in general practice or geriatrics.
- During out-of hours, the regular doctor is not available, instead out-of hours service doctors are responsible.

Sweden

Participating clusters consist of nursing homes with medical care provided by GPs. Nursing homes are reserved for the most vulnerable older persons, those who need 24 hours surveillance and/or are severely dependent in ADL.

- Medical care is provided by GPs. Sometimes, more than one GP (practice) is connected to the nursing homes. The GP practices are organised by regional authorities.
- During out-of hours, the regular GP/GP-practice will not be available, instead out-of hours service doctors are responsible.
- Nursing homes are organised by municipalities (separate from the regional authorities). Care is provided by nurse assistants (24-7 service) at the nursing homes. Nurses are available 24-7 but not always present at the nursing homes, as a nurse will be responsible for several nursing homes during evening/nights and weekends.

Data supplement 2: Decision Tool

The decision tool (Van Buul et al. 2018) is the core of the intervention and assists in the decision to prescribe or not prescribe antibiotics. There is a separate tool for patients with and without urinary catheter.

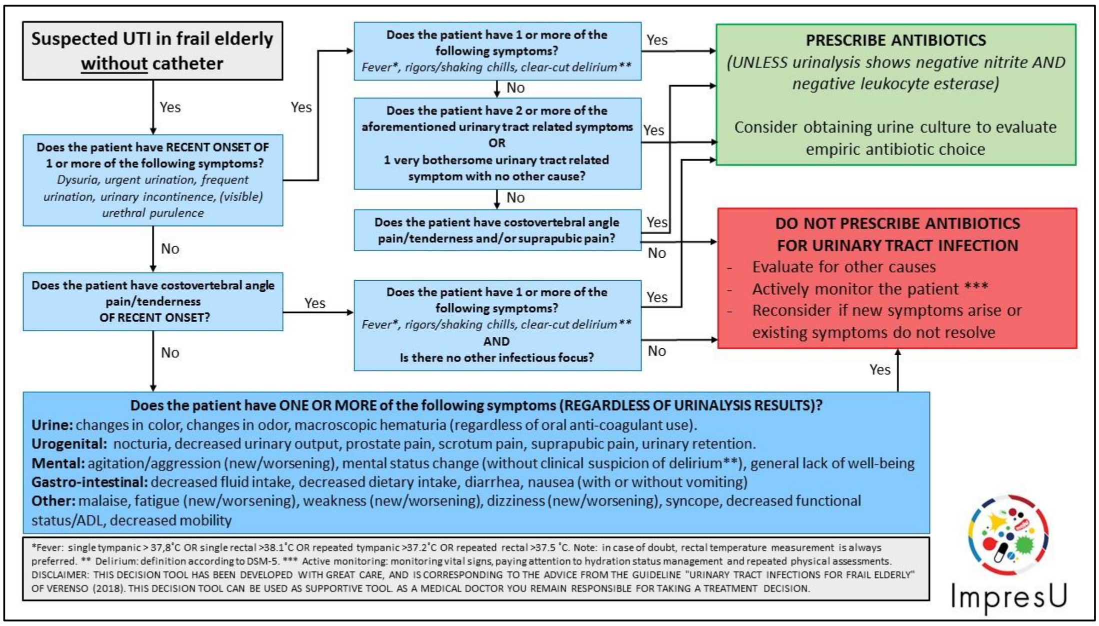

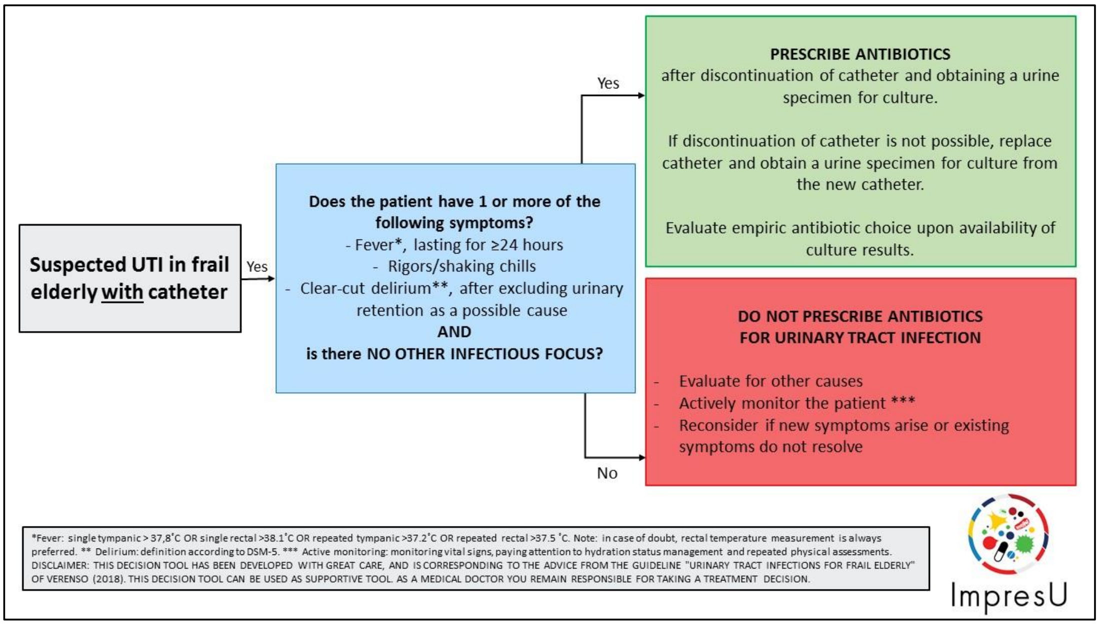

Reference: van Buul LW, Vreeken HL, Bradley SF, et al. The Development of a Decision Tool for the Empiric Treatment of Suspected Urinary Tract Infection in Frail Older Adults: A Delphi Consensus Procedure. J Am Med Dir Assoc 2018;19(9):757-64. doi: 10.1016/j.jamda.2018.05.001 [published Online First: 2018/06/19]

Data supplement 3: Example of toolbox materials

The pocket card for nursing staff is shown. It provides guidance of how to recognize a UTI, when to contact a doctor, and advice for an active monitoring policy. The pocket card is translated for each participating country, and may be tailored to the specific cluster.

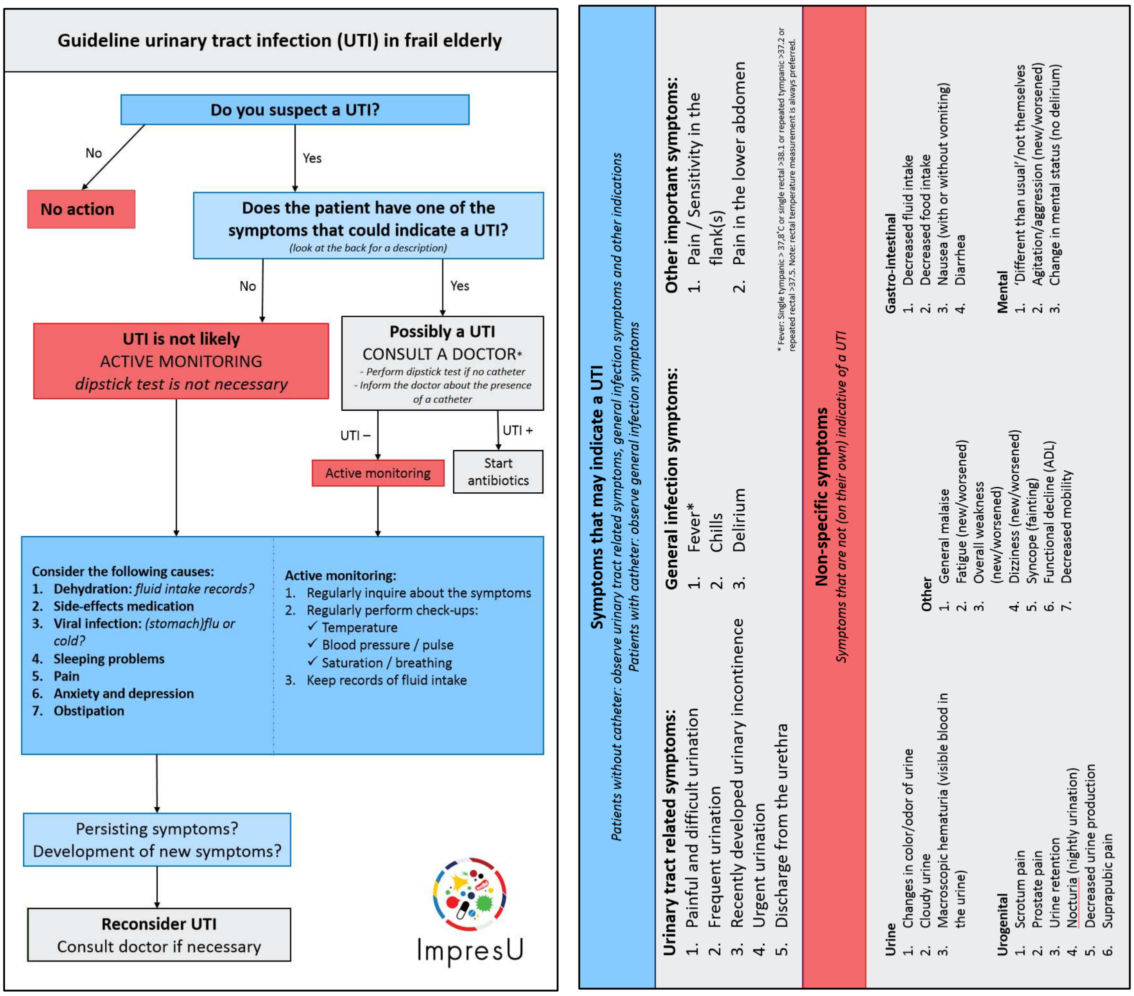

Supplement: Supplementary data [file bmjopen-2021-052552supp001.pdf]
