# Supplementary material for: Multifaceted antibiotic stewardship intervention using a participatory-action-research approach to improve antibiotic prescribing for urinary tract infections in frail elderly (ImpresU): study protocol for a European qualitative study followed by a pragmatic cluster randomised controlled trial
Source: BMJ Open. 2021 Oct 7;11(10):e052552. doi: 10.1136/bmjopen-2021-052552 (PMC8499316; doi:10.1136/bmjopen-2021-052552)
Supplement: Author's manuscript [file bmjopen-2021-052552.draft_revisions.pdf]

BMJ Open

BMJ Open is committed to open peer review. As part of this commitment we make the peer review history of every article we publish publicly available.

When an article is published we post the peer reviewers' comments and the authors' responses online. We also post the versions of the paper that were used during peer review. These are the versions that the peer review comments apply to.

The versions of the paper that follow are the versions that were submitted during the peer review process. They are not the versions of record or the final published versions. They should not be cited or distributed as the published version of this manuscript.

BMJ Open is an open access journal and the full, final, typeset and author-corrected version of record of the manuscript is available on our site with no access controls, subscription charges or pay-per-view fees (http://bmjopen.bmj.com).

If you have any questions on BMJ Open's open peer review process please email info.bmjopen@bmj.com

BMJ Open

A multifaceted antibiotic stewardship intervention using a participatory-action-research approach to improve antibiotic prescribing for urinary tract infections in frail elderly (ImpresU): study protocol for a European qualitative study followed by a pragmatic cluster randomised controlled trial.

Journal:	*BMJ Open*
Manuscript ID	bmjopen-2021-052552
Article Type:	Protocol
Date Submitted by the Author:	20-Apr-2021
Complete List of Authors:	Hartman, Esther; Amsterdam UMC Locatie VUmc, Medicine for older people; Utrecht University, Julius Center for Health Sciences and Primary Care, University Medical Center Utrecht<br>Groen, Wim; Amsterdam UMC Locatie VUmc, Medicine for older people<br>Heltveit-Olsen, Silje ; University of Oslo, The Antibiotic Centre for Primary Care, Department of General Practice, Institute of Health and Society<br>Lindbaek, Morten; University of Oslo, The Antibiotic Centre for Primary Care, Department of General Practice, Institute of Health and Society<br>Hoye, Sigurd; University of Oslo, The Antibiotic Centre for Primary Care, Department of General Practice, Institute of Health and Society<br>Sundvall, Pär-Daniel ; University of Gothenburg, General Practice/Family Medicine, School of Public Health and Community Medicine, Institute of Medicine, Sahlgrenska Academy; Region Västra Götaland, Research, Education, Development & Innovation, Primary Health Care<br>Gunnarsson, Ronny; University of Gothenburg, General Practice/Family Medicine, School of Public Health and Community Medicine, Institute of Medicine, Sahlgrenska Academy; Region Västra Götaland, Research, Education, Development & Innovation, Primary Health Care<br>Skoglund, Ingmarie; University of Gothenburg, General Practice/Family Medicine, School of Public Health and Community Medicine, Institute of Medicine, Sahlgrenska Academy; Region Västra Götaland, Research, Education, Development & Innovation, Primary Health Care<br>Snaebjörnsson Arnljots, Egill ; University of Gothenburg, General Practice/Family Medicine, School of Public Health and Community Medicine, Institute of Medicine, Sahlgrenska Academy; Region Västra Götaland, Research, Education, Development & Innovation, Primary Health Care<br>Godycki-Cwirko , Maciej; Medical University of Lodz, Centre for Family and Community Medicine, the Faculty of Health Sciences<br>Kowalczyk, Anna; Medical University of Lodz, Centre for Family and Community Medicine, the Faculty of Health Sciences<br>Platteel, Tamara ; Utrecht University, Julius Center for Health Sciences and Primary Care, University Medical Center Utrecht<br>Zuithoff, Nicolaas; Utrecht University, Julius Center for Health Sciences

and Primary Care, University Medical Center Utrecht
Monnier, Annelie; Amsterdam UMC Locatie VUmc, Medicine for older people
Verheij, Theo; Utrecht University, Julius Center for Health Sciences and Primary Care, University Medical Center Utrecht
Hertogh, Cees; Amsterdam UMC Locatie VUmc, Medicine for older people
van de Pol, Alma; Utrecht University, Julius Center for Health Sciences and Primary Care, University Medical Center Utrecht

Keywords: Urinary tract infections < UROLOGY, PRIMARY CARE, MEDICAL EDUCATION & TRAINING

SCHOLARONE™ Manuscripts

I, the Submitting Author has the right to grant and does grant on behalf of all authors of the Work (as defined in the below author licence), an exclusive licence and/or a non-exclusive licence for contributions from authors who are: i) UK Crown employees; ii) where BMJ has agreed a CC-BY licence shall apply, and/or iii) in accordance with the terms applicable for US Federal Government officers or employees acting as part of their official duties; on a worldwide, perpetual, irrevocable, royalty-free basis to BMJ Publishing Group Ltd ("BMJ") its licensees and where the relevant Journal is co-owned by BMJ to the co-owners of the Journal, to publish the Work in this journal and any other BMJ products and to exploit all rights, as set out in our licence.

The Submitting Author accepts and understands that any supply made under these terms is made by BMJ to the Submitting Author unless you are acting as an employee on behalf of your employer or a postgraduate student of an affiliated institution which is paying any applicable article publishing charge ("APC") for Open Access articles. Where the Submitting Author wishes to make the Work available on an Open Access basis (and intends to pay the relevant APC), the terms of reuse of such Open Access shall be governed by a Creative Commons licence – details of these licences and which Creative Commons licence will apply to this Work are set out in our licence referred to above.

Other than as permitted in any relevant BMJ Author's Self Archiving Policies, I confirm this Work has not been accepted for publication elsewhere, is not being considered for publication elsewhere and does not duplicate material already published. I confirm all authors consent to publication of this Work and authorise the granting of this licence.

For peer review only

TITLE

A multifaceted antibiotic stewardship intervention using a participatory-action-research approach to improve antibiotic prescribing for urinary tract infections in frail elderly (ImpresU): study protocol for a European qualitative study followed by a pragmatic cluster randomised controlled trial.

AUTHORS

Esther A.R. Hartman[1,2], Wim G. Groen[1,], Silje Rebekka Heltveit-Olsen[3], Morten Lindbæk[3], Sigurd Høye[3], Pär-Daniel Sundvall[4, 5], Ronny Gunnarsson[4, 5], Ingmarie Skoglund[4, 5] , Egill Snaebjörnsson Arnljots[4, 5], Maciej Godycki-Cwirko[6], Anna Kowalczyk[6], Tamara N. Platteel[2] , Nicolaas. P.A. Zuithoff[2], Annelie A. Monnier[1] Theo J.M. Verheij[2], Cees M.P.M. Hertogh[1] Alma C. van de Pol[2]

Corresponding author: Esther Hartman, Department of medicine for older people, Amsterdam UMC location VUmc, Room B-353, Van der Boechorstraat 7, 1081 BT, Amsterdam, e.hartman1@amsterdamumc.nl

Affiliations

[1] Department of medicine for older people, Amsterdam UMC location VUmc , the Netherlands
[2] Julius Center for Health Sciences and Primary Care, University Medical Center Utrecht, Utrecht University, the Netherlands.
[3] The Antibiotic Centre for Primary Care, Department of General Practice, Institute of Health and Society, University of Oslo, Norway
[4]General Practice/Family Medicine, School of Public Health and Community Medicine, Institute of Medicine, Sahlgrenska Academy, University of Gothenburg, Gothenburg, Sweden
[5]Research, Education, Development & Innovation, Primary Health Care, Region Västra Götaland, Sweden
[6] Centre for Family and Community Medicine, the Faculty of Health Sciences, the Medical University of Lodz, Poland

For peer review only

Word count: 3453

For peer review only - http://bmjopen.bmj.com/site/about/guidelines.xhtml

ABSTRACT

Introduction Almost 60% of antibiotics in frail elderly are prescribed for alleged urinary tract infections (UTIs). A substantial part of this comprises prescriptions in case of non-specific symptoms or asymptomatic bacteriuria, for which the latest guidelines promote restrictiveness with antibiotics. We aim to reduce inappropriate antibiotic use for UTIs through an antibiotic stewardship intervention (ASI) that encourages to prescribe according to these guidelines. To develop an effective ASI, we first need a better understanding of the complex decision-making process concerning suspected UTIs in frail elderly. Moreover, the implementation approach requires tailoring to the heterogeneous elderly care setting.

Methods and analysis First, we conduct a qualitative study to explore factors contributing to antibiotic prescribing for UTIs in frail elderly, using semi-structured interviews with general practitioners, nursing staff, patients, and informal caregivers. Next, we perform a pragmatic cluster randomised controlled trial in elderly care organisations. A multifaceted ASI is implemented in the intervention group; the control group receives care as usual. The ASI is centred around a decision tool that promotes restrictive antibiotic use, supported by a toolbox with educational materials. For the implementation, we use a modified participatory-action-research approach, guided by the results of the qualitative study. The primary outcome is the number of antibiotic prescriptions for suspected UTIs. We aim to recruit 34 clusters with in total 680 frail elderly residents ≥ 70 years. Data collection takes place during a 5-month baseline period and a 7-month follow-up period. Finally, we perform a process evaluation. The study has been delayed for 6 months due to COVID-19 and is expected to end in July 2021.

Ethics and dissemination Ethical approvals and/or waivers were obtained from the ethical committees in Poland, the Netherlands, Norway, and Sweden. The results will be disseminated through publication in peer-reviewed journals and conference presentations.

Trial Registration number NCT03970356

KEYWORDS

Urinary tract infections; Primary care; Medical education & Training

For peer review only - http://bmjopen.bmj.com/site/about/guidelines.xhtml

ARTICLE SUMMARY

Strengths and limitations of this study

- The qualitative study allows for a comprehensive analysis of the factors at play in decision-making on UTIs in frail elderly, which is essential to make progress in antibiotic stewardship in this setting.
- The pragmatic approach with its diverse international setting offers both broad applicability of results in general practice- and elderly care medicine, and gives a chance to evaluate country-specific outcomes.
- The use of participatory action research (PAR) embedded within a cluster randomised trial is infrequent, and may offer valuable insights for future trials; however, a limitation of the tailored approach is that the results will not be exactly replicable.
- The process evaluation of the PAR approach will provide guidance for implementation in daily practice, including a toolbox with supportive educational materials.
- The COVID-19 pandemic began in the midst of the implementation process, undoubtedly affecting the process and results.

For peer review only - http://bmjopen.bmj.com/site/about/guidelines.xhtml

INTRODUCTION

Background and rationale

Suspected urinary tract infections (UTIs) account for the majority of antibiotic prescriptions in frail elderly; it is estimated that between 32 and 62% of these are inappropriately prescribed to patients with only non-specific symptoms [1, 2](Sundvall, NAPCRG conference 2017, unpublished). In recent years, international efforts have been made to improve appropriate antibiotic prescribing: a decision tool to support physician's prescribing decisions was developed [3], and recent guidelines promote restrictive antibiotic use for UTIs in frail elderly [4]. However, international evidence from a randomised controlled trial on their efficacy in reducing inappropriate antibiotic use for UTIs is currently lacking.

Antibiotic prescribing decisions are known to be complex and influenced by many social and organisational factors [5, 6]. In UTIs in frail elderly, this is further complicated by diagnostic uncertainties. First frail elderly patients often present with non-specific symptoms. These symptoms should be evaluated for other causes but are often directly attributed to UTIs [2-4, 7, 8]. Second, interpretation of urinalysis is clouded by the high prevalence of asymptomatic bacteriuria, for which antibiotics are not needed [4, 7]. A rigorous behavioural change is required from multiple health care professionals to improve antibiotic prescribing in this population. In order to develop effective antibiotic stewardship interventions (ASIs), it is essential to better understand the complex process leading to the decision to (not) prescribe antibiotics for alleged UTIs. Given the large variety in the organisation of elderly care, it is unlikely that a uniform ASI is effective [9]. Participatory action research (PAR) is a promising method that actively involves the health care professionals to implement an ASI tailored to their setting, while accounting for local barriers and facilitators [10].

We set out to evaluate whether a multifaceted ASI is effective in reducing antibiotic prescribing for UTIs in frail elderly in various long-term care settings (in Poland, the Netherlands, Norway, and Sweden). To accomplish the substantial behavioural changes that are needed, we believe we need a combination of qualitative methods for exploration and a PAR approach for implementation. First, we perform a qualitative study with semi-structured interviews to develop a conceptual model of factors contributing to antibiotic prescribing decisions in this population. Then we conduct a cluster randomised controlled trial (RCT) in frail elderly in care homes attended by general practitioners (GPs) using PAR to implement an ASI. Finally, we conduct a process evaluation.

Objectives

- Obtain insights into all relevant factors that contribute to antibiotic prescribing for UTIs in frail elderly.

For peer review only - http://bmjopen.bmj.com/site/about/guidelines.xhtml

- Develop a conceptual model integrating these identified factors to guide the development of ASI for UTIs in frail elderly.
- Study the effects of the implementation of a multifaceted ASI on antibiotic prescription rates for UTIs in frail elderly.
- Evaluate the implementation process to understand the cluster RCT outcomes, and the added value of the PAR approach to implement ASIs.

METHODS AND ANALYSIS

The Improving antibiotic Prescribing for Urinary tract infections in frail elderly (ImpresU) study consists of a qualitative study and a cluster RCT. Their integration is shown in figure 1.

Qualitative study

The aims are to explore all relevant factors that contribute to antibiotic (non-)prescribing for UTIs in frail elderly, and to integrate these into a conceptual model to guide the development of effective ASIs.

Design and setting

An exploratory qualitative study using semi-structured interviews is conducted in Poland, the Netherlands, Norway, and Sweden. Interviews are conducted with representatives of three relevant stakeholder groups in the setting of elderly care at home and in institutions: 1) GPs, 2) nursing staff, and 3) patients and informal caregivers.

Eligibility criteria, recruitment and sample size

Recruitment takes place through the networks of the research teams per country. Variation is aspired within the representatives of each stakeholder group (e.g. in years of experience for health care professionals/workers). All participants need to be capable and willing to provide informed consent and communicate personal thoughts in the local language. Patients need to be 70 years or older, and are not recruited during the acute phase of a disease. The aim is to conduct approximately 60 interviews (i.e. 15 per country), preferably equally distributed over the three stakeholder groups.

Data collection and management

Topic lists and interview guides are designed based on literature [6] and (clinical) experience from the researchers. Pilot interviews are performed in each country to verify the appropriateness and completeness of the topic lists. All interviews are conducted in the native language and audio-recorded. Basic demographic data (e.g., gender, age) of participants are collected. Collected data and transcripts are pseudonymised, using a code for each participant.

For peer review only - http://bmjopen.bmj.com/site/about/guidelines.xhtml

Data analysis

Data are analysed with use of the framework method [11], which consists of the following steps: 1) Interviews are transcribed verbatim and translated into English. 2) The researchers (re)read the interviews for familiarisation. 3) Two researchers independently code a first batch of interviews. 4) Through consensus, a preliminary framework is formed. 5) The remaining interviews are coded using the framework; additions and changes are discussed within the research team. 6) Data are organised in a framework matrix. 7) Data are interpreted, and a conceptual model of factors is derived from the matrix.

Cluster randomised controlled trial

The trial aims to evaluate whether a decision tool for restrictive antibiotic use, implemented using a PAR-approach, reduces antibiotic prescribing for UTIs in frail elderly. For this report, we used the SPIRIT reporting guidelines [12].

Design and setting

A cluster RCT is performed in nursing homes in Poland, the Netherlands, Norway and Sweden, and in residential care homes and home care organisations in the Netherlands, attended by GPs. More details on the setting are provided in the Data Supplement 1. The cluster and unit of randomisation is the care organisation linked to the GP practice; one care organisation may be attended by multiple GP practices or vice versa. In the final months of the study period, a process evaluation is performed.

Eligibility criteria and recruitment

Recruitment of clusters is performed through the networks of the research groups in Poland, the Netherlands, Norway and Sweden. The care organisations identify eligible patients, provide written study information, and ask whether they may be approached by the research team. Written informed consent from patients (or representatives in case of legal incapacity) is obtained by a visiting researcher or nurse.

For inclusion, patients need to be 70 years or older, have physical and/or mental disabilities and ADL dependency requiring care, do not use prophylactic antibiotics, do not receive hospice care and are estimated not to have a very limited life expectancy (≤1 month). Patients are excluded when they start prophylactic antibiotics, start receiving hospice care, have a limited life expectancy (≤1 month), pass away, or move away from the cluster. Patients need to be included for at least two months to contribute data to the study.

Sample size

The baseline incidence of UTI prescriptions is assumed to be 0.75 per patient-year [13-16]. It has been shown that between 32% and 62% of these prescriptions are inappropriate, i.e. not

For peer review only - http://bmjopen.bmj.com/site/about/guidelines.xhtml

based on specific signs and symptoms [1, 2](Sundvall NAPCRG conference 2017, unpublished). After implementation of the algorithm, we assume the prescription rate to be reduced from 0.75 to 0.4 prescriptions per person-year. The intracluster correlation coefficient (ICC) is expected to be 0.06, in line with related studies in the primary care and nursing home setting [17, 18].

For the sample size calculation, a Wilcoxon Test with an adjustment for cluster randomisation was performed. With an expected cluster size of 10 patients, each contributing 7 months in the follow-up period, one-sided testing, alpha of 0.05, and power of 0.8, it is estimated that 333 patients are needed, translating into a minimum of 34 clusters. To compensate for loss to follow-up, we assume 20 patients per cluster are needed. In sum, we aim to include 34 participating clusters, i.e. 9 in each country, with in total 680 patients.

Randomisation and blinding

Clusters are randomised to intervention or usual care, using SAS software v9.4 [19] by an independent data manager. Block randomisation is used to assign clusters to intervention or control in each country, stratified on cluster size (small/medium/large). Due to the nature of the intervention, blinding is not possible; however, the aims of the study outcomes are not explicitly stated to the control clusters to avoid contamination.

Intervention

The intervention clusters receive a multifaceted ASI. The control clusters provide care as usual. The intervention period was intended to last 4 months. After a month, it was interrupted by the first wave of the COVID-19 pandemic, resulting in a 6-month pause. Upon restart in September 2020, the pragmatic choice was made to restart the intervention period with a duration of 2-3 months, depending on the local situation.

Decision tool & Toolbox

At the core of the ASI is a decision tool to guide the use of antibiotics for suspected UTIs in frail elderly [3] (Data Supplement 2). It promotes an active monitoring approach in case only nonspecific symptoms are present. This decision tool is incorporated in the Dutch UTI guideline for elderly care medicine and congruent with the Swedish and Norwegian UTI guidelines [20-22]. To support the implementation of the decision tool, a toolbox of educational materials is composed (Figure 2 and Data Supplement 3). First a generic toolbox is designed, centred around the decision tool. Next, it is tailored to become country-specific by the local researchers, based on the qualitative study data and any locally available materials. During the intervention period, further tailoring may take place within the participating cluster itself (Figure 2).

Implementation: modified PAR approach

For peer review only - http://bmjopen.bmj.com/site/about/guidelines.xhtml

The intervention is tailored based on an analysis of the interview data to identify country-specific barriers and facilitators. For example, the roles of the health care professionals and knowledge gaps in care for UTIs differ per country and need to be targeted accordingly. During the intervention period, the researchers and health care professionals together go through a cyclical process of reflection, planning and action during sessions for education and evaluation. These sessions combine a top-down and bottom-up approach; both education on the decision tool and any knowledge gaps identified in the qualitative study, as well as reflection and planning for local implementation. The aim is to go through at least two PAR cycles in each cluster, and to actively involve physicians as well as nursing staff. Further tailoring may be performed in each country and cluster locally.

Outcome assessments

Primary outcome measure:

1. Number of prescriptions of antibiotics for suspected UTIs

Secondary outcome measures:

2. Number of prescriptions of antibiotics for suspected UTIs in office hours
3. Number of incorrect prescriptions of antibiotics for suspected UTIs
4. Incidence of suspected UTIs
5. Incidence of complications within 21 days after each UTI suspicion (presence yes/no of a complication: delirium, pyelonephritis, sepsis and renal failure)
6. Incidence of referral to a hospital within 21 days after each UTI suspicion
7. Incidence of hospital admission within 21 days after each UTI suspicion
8. Mortality
9. Mortality within 21 days after each UTI suspicion

All outcomes are assessed during the follow-up period, and expressed per patient-year.

Data collection

Data are collected during a 5-month baseline period and a 7-month follow-up period, through case report forms (CRFs) completed by the GP, nurse or researcher based on contact with a health care professional or medical file. The timeline for participating clusters and participants is displayed in figure 3.

For each participant, a CRF with patient characteristics is filled in at study entry consisting of items concerning demographics, ADL-dependency measured through the Katz Index of Independence in Activities of Daily Living [23], and relevant medical history. The GPs prospectively register each UTI suspicion on a short registration form, describing symptoms, diagnostics, and antibiotic treatment (primary and secondary outcomes). After 7 and 21 days, follow-up forms are filled in to assess the course of disease, any change in antibiotic treatment, complications, and mortality (primary and secondary outcomes). Overall mortality (secondary outcome) is registered upon exclusion of a patient. Any missing data

For peer review only - http://bmjopen.bmj.com/site/about/guidelines.xhtml

are retrospectively registered through consultation of GPs, nurses and/or access of the medical records.

Furthermore, anonymised data concerning COVID-19 incidence in the participating care organisations is registered during the follow-up period.

Data management

Data are collected pseudonymised on paper forms, using a study code for each patient. Afterwards, they are electronically registered in the secured online database Research Online, according to ICH-GCP regulations. Research Online has multiple validation rules built into the eCRFs. The data cleaning process is supported by automatically and manually generated queries. At the end of the study, all data will be locked. Dedicated data sets are provided to the researchers for analysis. Data are kept securely for at least 15 years.

Data analysis

The analysis will follow the intention-to-treat principle. For the primary outcome, a generalised linear mixed model for Poisson distributions will be used. In case the assumptions for Poisson distributions are insufficiently met, other distributions will be considered (i.e. negative binomial, generalised Poisson, zero-inflated Poisson). A random intercept will be included to correct for clustering within care facility and/or GP, and an additional random intercept will be included to correct for repeated measurements in patients. When results indicate no or very low clustering at the facility/GP or patient level, the corresponding random intercept will be excluded from the analysis. The comparison between intervention and control group, estimated with the time by treatment interaction, will be reported as Rate Ratio's with a 95% CI and a corresponding p-value. In a second model, pre-specified prognostic factors will be added: age, gender, ADL-dependency, presence of an indwelling catheter, dementia, recurrent UTIs, diabetes mellitus, and kidney disorders. In case there are missing values on baseline variables that were selected as potential confounders, multiple imputation will be considered. Furthermore, subgroup analysis will be performed to assess outcomes in groups per country, with different gender, age, presence of dementia, urinary incontinence, and indwelling catheter.

Process evaluation

A process evaluation is conducted in the care organisations participating in the cluster RCT. The framework described by Saunders et al. is used [24]. Elements that are assessed include fidelity, dose delivered/received, reach, recruitment, and context (including COVID-19 impact). Data are collected through documentation of the intervention process by the researchers, and through questionnaires with closed- and open-ended questions to participating health care personnel. Quantitative data will be reported using descriptive statistics; thematic analysis will be performed on the qualitative data.

For peer review only - http://bmjopen.bmj.com/site/about/guidelines.xhtml

DISCUSSION

We perform a European qualitative study exploring factors influencing decision making on UTIs in frail elderly, and a pragmatic cluster RCT to assess the effect of a decision tool to improve antibiotic prescribing for UTIs in frail elderly, implemented using a PAR-approach. We believe this combination of methodologies is essential to address the complexity of decision making on UTIs in this population. Drawing lessons from the IMPACT study [25], we are the first to apply this in a diverse international setting.

The PAR approach for implementation allows us to embrace the heterogeneity of the elderly care settings within and between countries [26]. With large-scale nursing homes in some countries and small-scale living facilities in others, an identical ASI for each health care professional will not be effective. Tailoring the intervention using PAR promotes bottom-up engagement of health care professionals, thereby enabling the required behavioural changes for lasting effects.

Inherent to the tailored approach are limits in the ability to exactly replicate our results. Nevertheless, the methods are replicable, and we believe our results will be widely applicable. The qualitative study will offer in-depth understanding of the factors involved in decisions on UTI, thereby creating opportunities for future ASI development. Our robust trial design, in line with epidemiological recommendations for evaluating ASI [27], will provide evidence on the application of the latest UTI guidelines. Furthermore, our process evaluation will generate understanding on the ASI and its components in the various settings, and will provide lessons on the use of PAR in future trials. Finally, a practical implementation package will become available, with relevant toolbox materials and lessons for daily practice to be tailored to any setting.

The cluster RCT was interrupted by the first wave of the COVID-19 pandemic during the intervention period, and was forced to pause for 6 months. Restarting required much flexibility from the participating care organisations, where patient care already suffered from the pandemic. Sessions for the intervention meeting had to be repeated (mostly online). Furthermore, the 6-month delay and further COVID-19 waves regrettably continue to lead to the passing away of participants, increasing the need for new recruitment. As randomisation takes place per country, we presume effects of COVID-19 on our population characteristics and outcomes, if any, will be balanced between intervention and control clusters.

In conclusion, we aim to evaluate the effectiveness of a multifaceted ASI to reduce antibiotic prescribing for UTIs in frail elderly through a qualitative study and cluster RCT in Poland, the Netherlands, Norway and Sweden. Our tailored approach within the diverse setting is promising to yield broadly applicable results, even if currently challenged by the COVID-19 pandemic.

For peer review only - http://bmjopen.bmj.com/site/about/guidelines.xhtml

ETHICS AND DISSEMINATION

Participant safety and monitoring

The cluster RCT is considered low risk, as the intervention corresponds to current guidelines. There is no data monitoring committee, and any SAEs are not reported. No interim analyses are planned. For both the qualitative study and cluster RCT respectively, ethical approval was given by the Committee of Bioethics of the Medical University of Lodz, Poland (RNN/381/18/KE and RNN/260/19/KE), the Regional Committee for Medical and Health Research Ethics in Norway (2018/2191/REK sør-øst A and 2018/2521/REK sør-øst A), and the Swedish Ethical Review Authority (2019-00504 and 2019-00796/1228-18(2019-02541)). In the Netherlands, the Medical Ethics Review Committees established that approval was not required since the Medical Research Involving Human Subjects Act does not apply (2018.500 VU University Medical Centre and WAG/mb/19/012207 University Medical Centre Utrecht). Substantial protocol modifications are communicated to ethical committees and the trial register. Dissemination will take place through publication and presentations. Furthermore, an implementation package will be developed.

Patient and public involvement

In the qualitative study, patients and informal caregivers are interviewed. These data were taken into account in the intervention implementation in the cluster RCT. In the process of the design of the cluster RCT, a meeting was held with representatives of Network Utrecht, care for the elderly (NUZO), Julius Centre, University Medical Centre Utrecht, the Netherlands. Their suggestions on the protocol were taken into account; for example, on patient-directed toolbox materials.

Trial Status

Currently, the cluster RCT is ongoing and expected to finish in July 2021. Database lock will take place in September 2021.

Author contributions

CH, TV, ML, PS, MGC conceptualised the study and obtained funding. For the qualitative study, AM drafted the protocol with EH, WG, SHO, ML, SH, PS, IS, ESA, AK, MGC and CH. For the cluster RCT, AP drafted the protocol with EH, WG, SHO, ML, SH, PS, RG, ESA, MGC, AK, TP, NZ, TV, and CH. NZ wrote the statistical analysis plan with EH and AP. EH, WG, AP, AM, TP, SHO, SH, AK, ESA and PS designed the process evaluation. The manuscript was drafted by EH and critically revised by all authors. All authors read and approved the final manuscript.

Acknowledgements

We wish to thank Sofia Sundvall and Sara Sofia Lithén, research nurses, for their ongoing efforts in data collection. Furthermore, we would like to express our gratitude to the participating general practices and elderly care organisations for their prolonged contributions despite the current pandemic.

For peer review only - http://bmjopen.bmj.com/site/about/guidelines.xhtml

Funding statement

This work was supported by JPI AMR with reference number JPIAMR_2017_P007, through national funding agencies: National Science Centre Poland (UMO-2017/25/Z/NZ7/03024), ZonMw the Netherlands(549003002), the Research Council of Norway (284253/H10), and The Swedish Research Council (2017-05975). The Healthcare Board, Region Västra Götaland (N/A) partially funded the Swedish part of the study. The funders have no role in or authority on study design, data collection, management, analysis or interpretation, writing and submission of reports for publication.

Competing interests statement

None declared

FULL REFERENCES

1. van Buul LW, Veenhuizen RB, Achterberg WP, et al. Antibiotic prescribing in Dutch nursing homes: how appropriate is it? *J Am Med Dir Assoc* 2015;16(3):229-37. doi: 10.1016/j.jamda.2014.10.003 [published Online First: 2014/12/03]
2. Mayne S, Sundvall PD, Gunnarsson R. Confusion Strongly Associated with Antibiotic Prescribing Due to Suspected Urinary Tract Infections in Nursing Homes. *J Am Geriatr Soc* 2018;66(2):274-81. doi: 10.1111/jgs.15179 [published Online First: 2018/01/11]
3. van Buul LW, Vreeken HL, Bradley SF, et al. The Development of a Decision Tool for the Empiric Treatment of Suspected Urinary Tract Infection in Frail Older Adults: A Delphi Consensus Procedure. *J Am Med Dir Assoc* 2018;19(9):757-64. doi: 10.1016/j.jamda.2018.05.001 [published Online First: 2018/06/19]
4. Nicolle LE, Gupta K, Bradley SF, et al. Clinical Practice Guideline for the Management of Asymptomatic Bacteriuria: 2019 Update by the Infectious Diseases Society of America. *Clin Infect Dis* 2019;68(10):1611-15. doi: 10.1093/cid/ciz021 [published Online First: 2019/09/12]
5. Colliers A, Coenen S, Remmen R, et al. How do general practitioners and pharmacists experience antibiotic use in out-of-hours primary care? An exploratory qualitative interview study to inform a participatory action research project. *BMJ Open* 2018;8(9):e023154. doi: 10.1136/bmjopen-2018-023154 [published Online First: 2018/10/01]
6. van Buul LW, van der Steen JT, Doncker SM, et al. Factors influencing antibiotic prescribing in long-term care facilities: a qualitative in-depth study. *BMC Geriatr* 2014;14:136. doi: 10.1186/1471-2318-14-136 [published Online First: 2014/12/18]
7. Sundvall PD, Ulleryd P, Gunnarsson RK. Urine culture doubtful in determining etiology of diffuse symptoms among elderly individuals: a cross-sectional study of 32 nursing homes. *BMC Fam Pract* 2011;12:36. doi: 10.1186/1471-2296-12-36 [published Online First: 2011/05/20]
8. Mayne S, Bowden A, Sundvall PD, et al. The scientific evidence for a potential link between confusion and urinary tract infection in the elderly is still confusing - a systematic literature review. *BMC Geriatr* 2019;19(1):32. doi: 10.1186/s12877-019-1049-7 [published Online First: 2019/02/06]
9. Leykum LK, Pugh JA, Lanham HJ, et al. Implementation research design: integrating participatory action research into randomized controlled trials. *Implement Sci* 2009;4:69. doi: 10.1186/1748-5908-4-69 [published Online First: 2009/10/27]
10. van Buul LW, Sikkens JJ, van Agtmael MA, et al. Participatory action research in antimicrobial stewardship: a novel approach to improving antimicrobial prescribing in hospitals and long-term care facilities. *J Antimicrob Chemother* 2014;69(7):1734-41. doi: 10.1093/jac/dku068 [published Online First: 2014/03/22]

For peer review only - http://bmjopen.bmj.com/site/about/guidelines.xhtml

11. Gale NK, Heath G, Cameron E, et al. Using the framework method for the analysis of qualitative data in multi-disciplinary health research. *BMC Med Res Methodol* 2013;13:117. doi: 10.1186/1471-2288-13-117 [published Online First: 2013/09/21]
12. Chan AW, Tetzlaff JM, Gotzsche PC, et al. SPIRIT 2013 explanation and elaboration: guidance for protocols of clinical trials. *BMJ* 2013;346:e7586. doi: 10.1136/bmj.e7586 [published Online First: 2013/01/11]
13. Resultaten van wekelijkse surveillance, referentiecijfers 2011-2015. Surveillance Netwerk Infectieziekten in Verpleeghuizen: Dutch National Institute for Public Health and the Environment.
14. Fagan M, Maehlen M, Lindbaek M, et al. Antibiotic prescribing in nursing homes in an area with low prevalence of antibiotic resistance: compliance with national guidelines. *Scand J Prim Health Care* 2012;30(1):10-5. doi: 10.3109/02813432.2011.629156 [published Online First: 2011/12/23]
15. Ricchizzi E, Latour K, Karki T, et al. Antimicrobial use in European long-term care facilities: results from the third point prevalence survey of healthcare-associated infections and antimicrobial use, 2016 to 2017. *Euro Surveill* 2018;23(46) doi: 10.2807/1560-7917.ES.2018.23.46.1800394 [published Online First: 2018/11/22]
16. Sundvall PD, Stuart B, Davis M, et al. Antibiotic use in the care home setting: a retrospective cohort study analysing routine data. *BMC Geriatr* 2015;15:71. doi: 10.1186/s12877-015-0073-5 [published Online First: 2015/06/26]
17. Campbell MK, Fayers PM, Grimshaw JM. Determinants of the intracluster correlation coefficient in cluster randomized trials: the case of implementation research. *Clin Trials* 2005;2(2):99-107. doi: 10.1191/1740774505cn071oa [published Online First: 2005/11/11]
18. Rutten JJS, van Buul LW, Smalbrugge M, et al. Antibiotic prescribing and non-prescribing in nursing home residents with signs and symptoms ascribed to urinary tract infection (ANNA): study protocol for a cluster randomized controlled trial. *BMC Geriatr* 2020;20(1):341. doi: 10.1186/s12877-020-01662-0 [published Online First: 2020/09/12]
19. SAS Version 9.4 [program]: SAS Institute Inc. SAS and all other SAS Institute Inc. product or service names are registered trademarks or trademarks of SAS Institute Inc., Cary, NC, USA.
20. Läkemedelsbehandling av urinvägsinfektioner i öppenvård - behandlingsrekommendation: Läkemedelsverket, 2017:21-36.
21. Richtlijn Urineweginfecties bij kwetsbare ouderen: Verenso, 2018.
22. Akselsen PEO, S. Urinveisinfeksjoner i sykehjem, versjon 2.2: Antibiotikasenteret for primærmedisin, 2020.
23. Katz S, Ford AB, Moskowitz RW, et al. Studies of Illness in the Aged. The Index of Adl: A Standardized Measure of Biological and Psychosocial Function. *JAMA* 1963;185:914-9. doi: 10.1001/jama.1963.03060120024016 [published Online First: 1963/09/21]
24. Saunders RP, Evans MH, Joshi P. Developing a process-evaluation plan for assessing health promotion program implementation: a how-to guide. *Health Promot Pract* 2005;6(2):134-47. doi: 10.1177/1524839904273387 [published Online First: 2005/04/28]
25. van Buul LW, van der Steen JT, Achterberg WP, et al. Effect of tailored antibiotic stewardship programmes on the appropriateness of antibiotic prescribing in nursing homes. *J Antimicrob Chemother* 2015;70(7):2153-62. doi: 10.1093/jac/dkv051 [published Online First: 2015/03/07]
26. Van Buul LW, Monnier AA, Sundvall PD, et al. Antibiotic Stewardship in European Nursing Homes: Experiences From the Netherlands, Norway, Poland, and Sweden. *J Am Med Dir Assoc* 2020;21(1):34-40 e1. doi: 10.1016/j.jamda.2019.10.005 [published Online First: 2019/12/04]
27. de Kraker MEA, Abbas M, Huttner B, et al. Good epidemiological practice: a narrative review of appropriate scientific methods to evaluate the impact of antimicrobial stewardship interventions. *Clin Microbiol Infect* 2017;23(11):819-25. doi: 10.1016/j.cmi.2017.05.019 [published Online First: 2017/06/03]

For peer review only - http://bmjopen.bmj.com/site/about/guidelines.xhtml

FIGURE LEGENDS

Figure 1: Schematic overview of the interplay between the two studies.
The qualitative study offers insights to tailor the antibiotic stewardship intervention in the cluster randomised controlled trial (RCT), through a country-specific local analysis. The cluster RCT consists of a baseline- and follow-up period for data collection, with an intervention period or usual care in between (the timeline is provided in Figure 3). A process evaluation follows at the end of the cluster RCT.

Figure 2: Toolbox.
The educational materials and targeted stakeholders in the generic toolbox are listed, and the tailoring process is shown.

Figure 3: Timeline of the cluster randomised controlled trial.
The periods of data collection and procedures are shown for the clusters and participating patients.

For peer review only

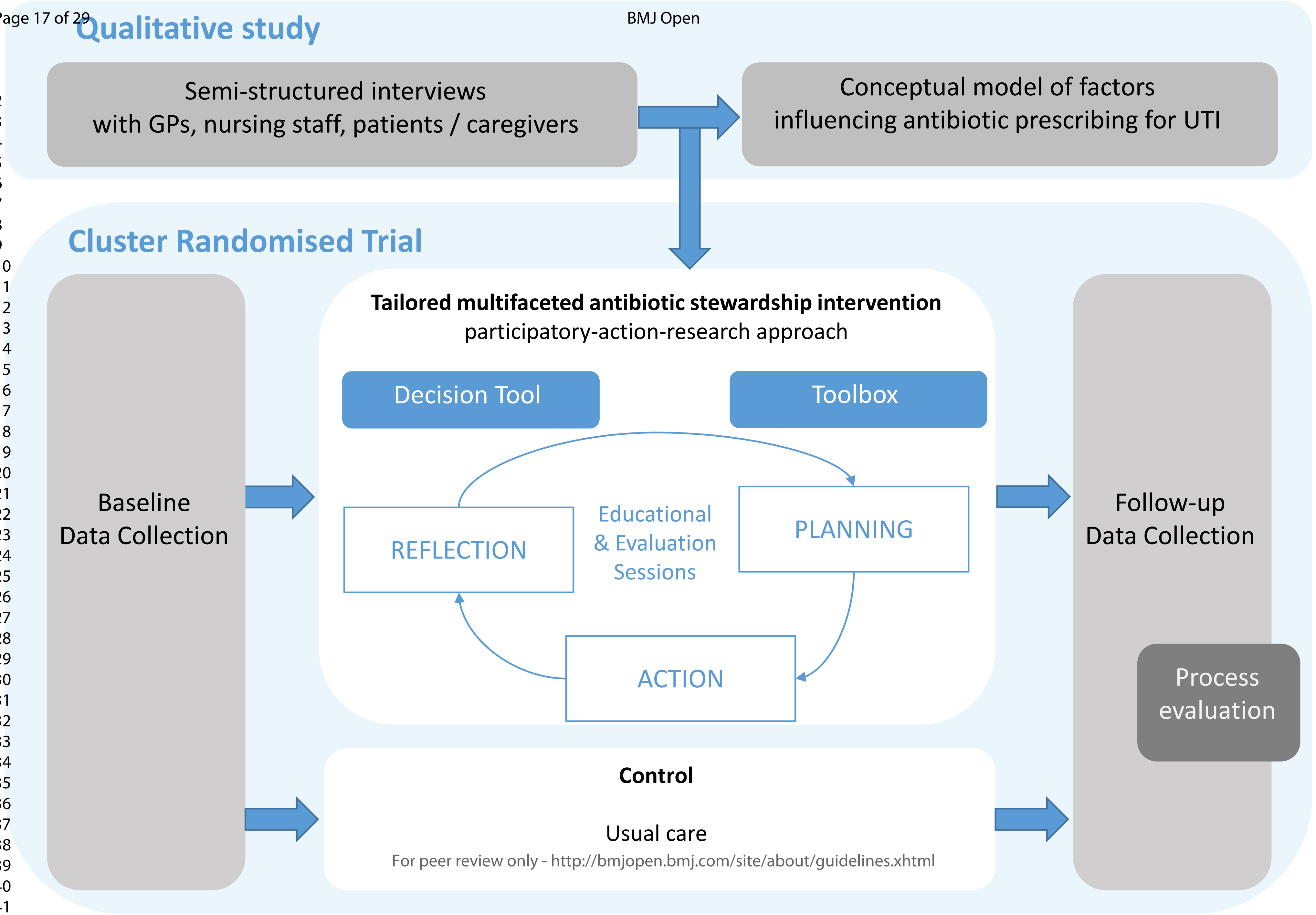

For peer review only - http://bmjopen.bmj.com/site/about/guidelines.xhtml

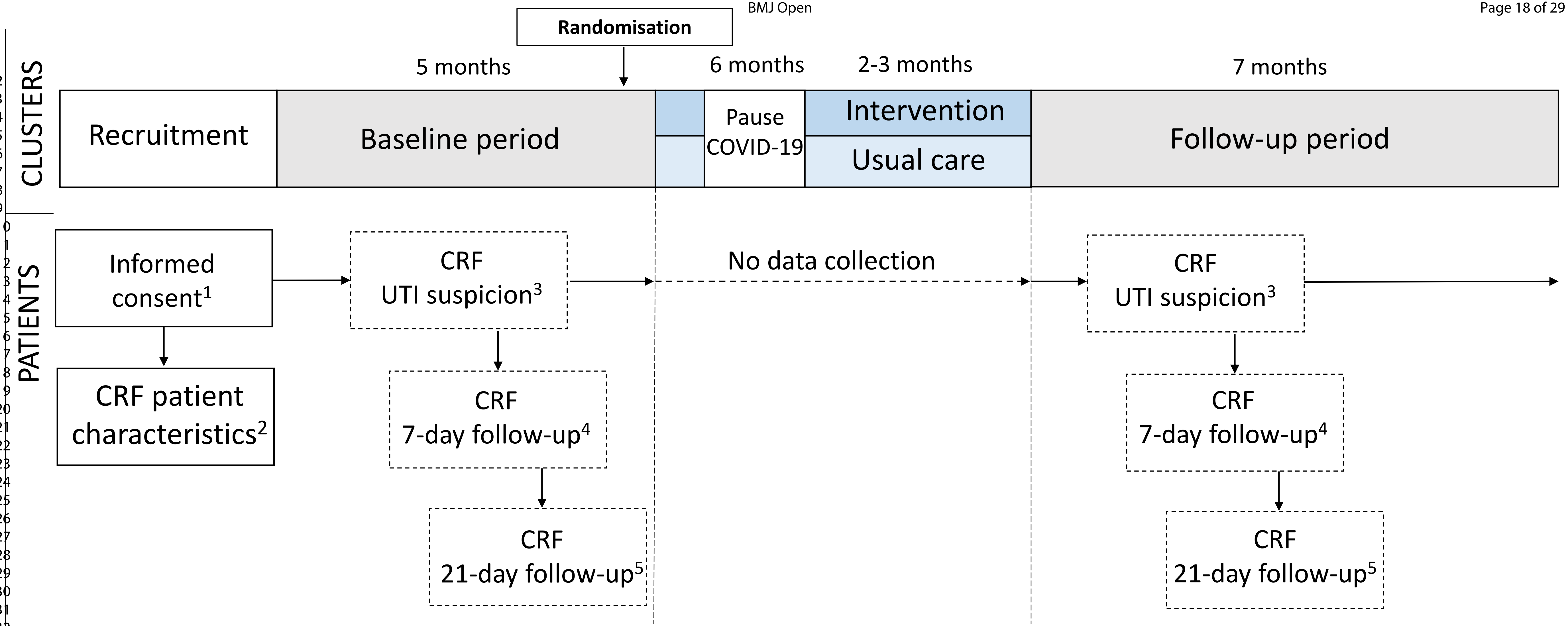

[1] Continuous enrollment throughout the study
[2] Demographics, ADL-dependency, relevant medical history
[3] Symptoms, diagnostics, antibiotic treatment
[4&5] Course of disease and treatment, complications, mortality

For peer review only - http://bmjopen.bmj.com/site/about/guidelines.xhtml

Materials (targeted stakeholder)
Information leaflet (GPs, nursing staff, patients)
Pocket card (GPs, nursing staff)
Poster (nursing staff, patients)
Active monitoring checklist (nursing staff)
Case study (GPs, nursing staff)
E-learning (nursing staff)

Tailoring process

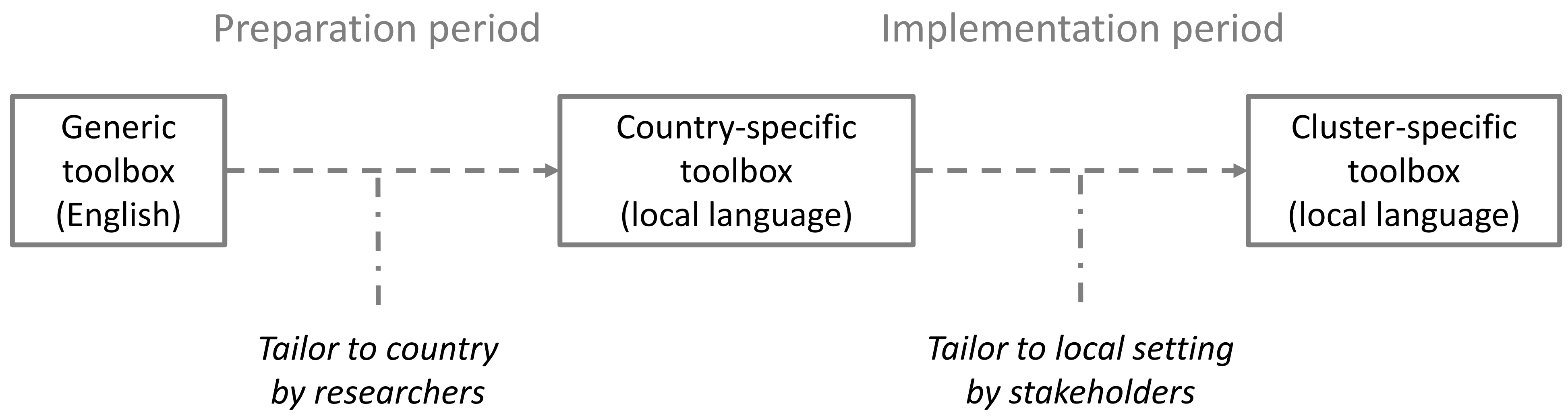

SUPPLEMENTARY DATA

Data supplement 1: Setting of the participating clusters in the cluster RCT

Poland

Participating clusters consist of nursing homes with patients registered to a particular GP primary care centre. Nursing homes in Poland provide living, care, support and educational services to people who require 24-hour care due to their age, illness or disability. Nursing homes may be conducted by local government units, churches, or other associations.

- Nursing care is provided 24-hours a day.
- Patients are registered to a particular GP in a primary care centre.
- Medical services are provided on the general principles of the National Health Fund. Patients can visit their GP in the centre or the GP comes to the nursing home on regular basis and on demand.
- During out-of hours, the regular GP/GP-practice is not be available. Instead, out-of hours service doctors are responsible or an ambulance is called in urgent cases.

The Netherlands

Participating clusters consist of residential care homes or home care organisation and their attending GP practices. This used to be a well-defined GP-attended setting; however, due to recent policy changes the setting is now quite heterogeneous. It does not include nursing homes; specialized elderly care physicians provide medical care in nursing homes.

- Patients receive varying degrees of ADL care, often provided by nurse-assistants with lower educational levels compared to the nursing home setting. Often, nurses are available (on-call). Patients may live in residential care homes or apartment complexes next to it, small-scale living facilities for dementia care, or have “regular” homes with access to home care.
- Medical care is provided by the GP. Often, more than one GP practice is connected to the nursing teams, as patients choose their own GP and their own nursing care organisation. In some residential care homes, the GP visits on a regular basis, for others, the GP is available only on demand.
- During out-of hours, the out-of-hour GP service is available instead of the regular GP.

For peer review only

Norway

Participating clusters consist of nursing homes with nursing home doctors providing medical care. Nursing homes are organised by municipalities, and are reserved for the most vulnerable older persons; those who need 24 hours surveillance and/or are severely dependent in ADL.

- 24-hour care is available at the nursing home from nurses and nurse assistants.
- Medical care is provided by nursing home doctors, with various medical backgrounds, e.g. in general practice or geriatrics.
- During out-of hours, the regular doctor is not available, instead out-of hours service doctors are responsible.

Sweden

Participating clusters consist of nursing homes with medical care provided by GPs. Nursing homes are reserved for the most vulnerable older persons, those who need 24 hours surveillance and/or are severely dependent in ADL.

- Medical care is provided by GPs. Sometimes, more than one GP (practice) is connected to the nursing homes. The GP practices are organised by regional authorities.
- During out-of hours, the regular GP/GP-practice will not be available, instead out-of hours service doctors are responsible.
- Nursing homes are organised by municipalities (separate from the regional authorities). Care is provided by nurse assistants (24-7 service) at the nursing homes. Nurses are available 24-7 but not always present at the nursing homes, as a nurse will be responsible for several nursing homes during evening/nights and weekends.

For peer review only

Data supplement 2: Decision Tool

The decision tool (Van Buul et al. 2018) is the core of the intervention and assists in the decision to prescribe or not prescribe antibiotics. There is a separate tool for patients with and without urinary catheter.

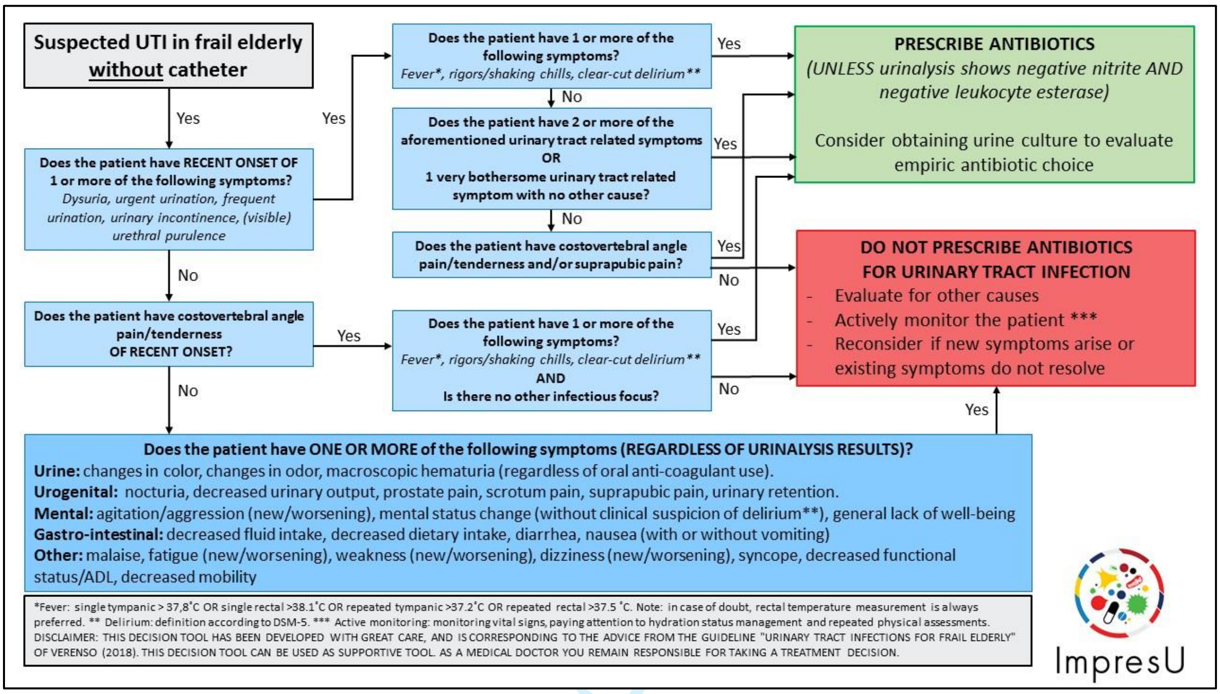

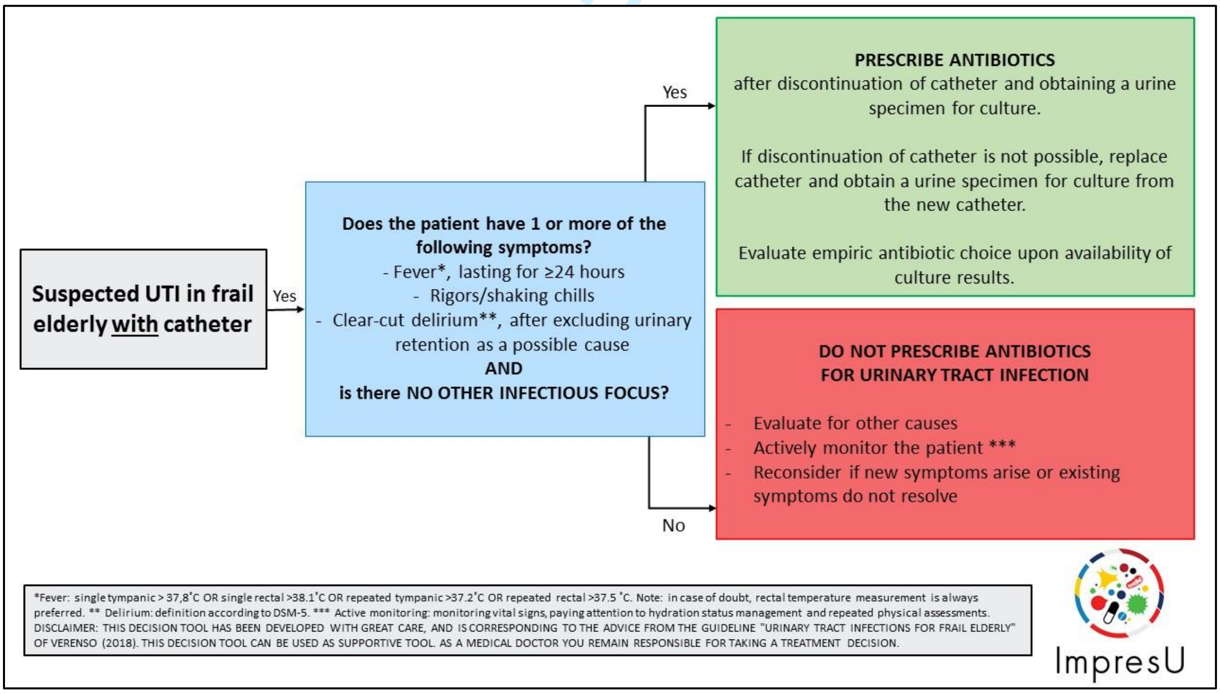

Reference: van Buul LW, Vreeken HL, Bradley SF, et al. The Development of a Decision Tool for the Empiric Treatment of Suspected Urinary Tract Infection in Frail Older Adults: A Delphi Consensus Procedure. J Am Med Dir Assoc 2018;19(9):757-64. doi: 10.1016/j.jamda.2018.05.001 [published Online First: 2018/06/19]

Data supplement 3: Example of toolbox materials

The pocket card for nursing staff is shown. It provides guidance of how to recognize a UTI, when to contact a doctor, and advice for an active monitoring policy. The pocket card is translated for each participating country, and may be tailored to the specific cluster.

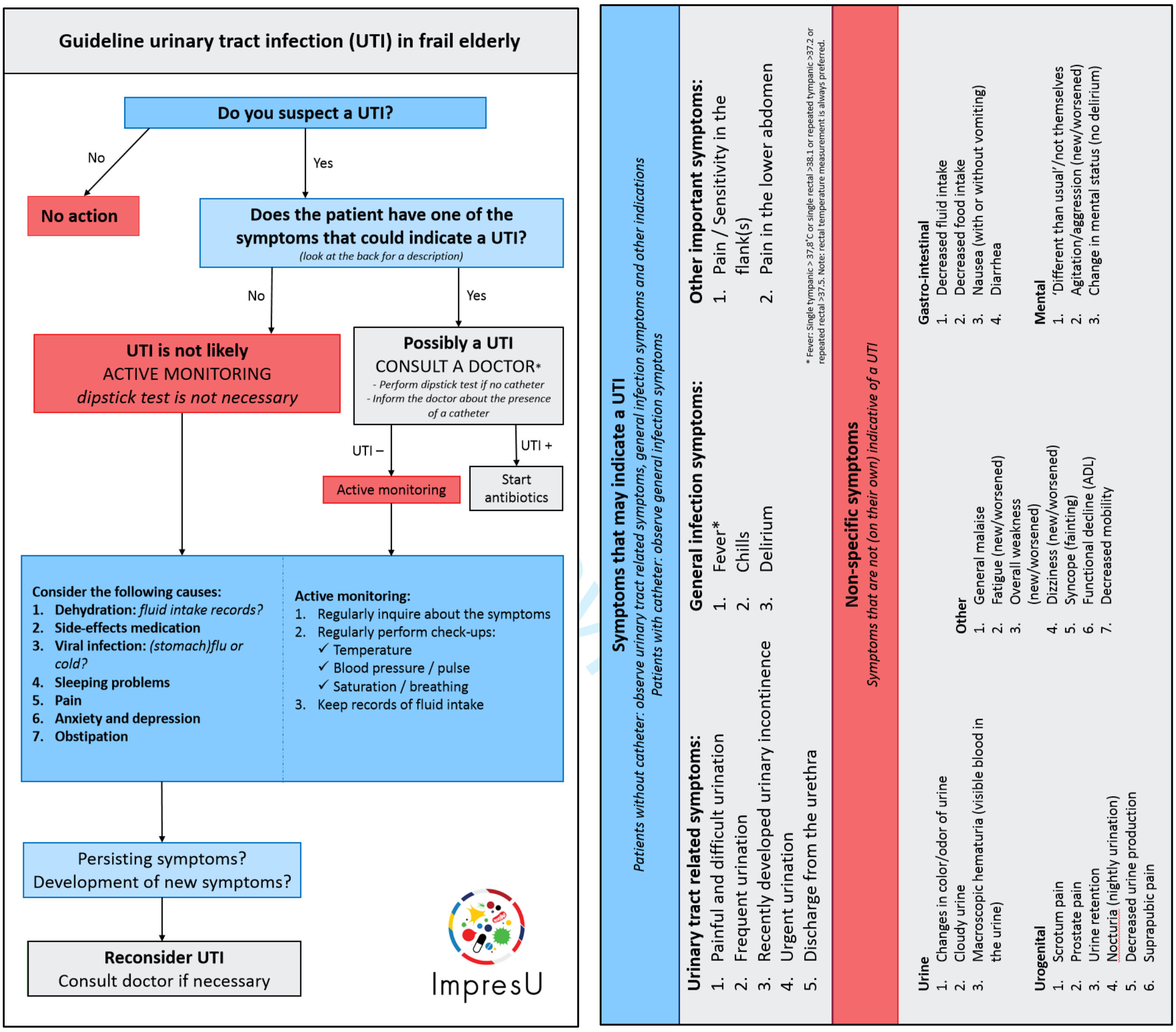

Reporting checklist for protocol of a clinical trial.

Based on the SPIRIT guidelines.

Instructions to authors

Complete this checklist by entering the page numbers from your manuscript where readers will find each of the items listed below.

Your article may not currently address all the items on the checklist. Please modify your text to include the missing information. If you are certain that an item does not apply, please write "n/a" and provide a short explanation.

Upload your completed checklist as an extra file when you submit to a journal.

In your methods section, say that you used the SPIRITreporting guidelines, and cite them as:

Chan A-W, Tetzlaff JM, Gøtzsche PC, Altman DG, Mann H, Berlin J, Dickersin K, Hróbjartsson A, Schulz KF, Parulekar WR, Krleža-Jerić K, Laupacis A, Moher D. SPIRIT 2013 Explanation and Elaboration: Guidance for protocols of clinical trials. BMJ. 2013;346:e7586

		Reporting Item	Page Number
**Administrative information**			
Title	#1	Descriptive title identifying the study design, population, interventions, and, if applicable, trial acronym	1
Trial registration	#2a	Trial identifier and registry name. If not yet registered, name of intended registry	2
Trial registration: data set	#2b	All items from the World Health Organization Trial Registration Data Set	2, NCT03970356
Protocol version	#3	Date and version identifier	V1.9 Dec 10, 2020
Funding	#4	Sources and types of financial, material, and other support	12

For peer review only

Roles and responsibilities: contributorship	#5a	Names, affiliations, and roles of protocol contributors	11
Roles and responsibilities: sponsor contact information	#5b	Name and contact information for the trial sponsor	n/a via corresponding author
Roles and responsibilities: sponsor and funder	#5c	Role of study sponsor and funders, if any, in study design; collection, management, analysis, and interpretation of data; writing of the report; and the decision to submit the report for publication, including whether they will have ultimate authority over any of these activities	12
Roles and responsibilities: committees	#5d	Composition, roles, and responsibilities of the coordinating centre, steering committee, endpoint adjudication committee, data management team, and other individuals or groups overseeing the trial, if applicable (see Item 21a for data monitoring committee)	11 n/a
**Introduction**			
Background and rationale	#6a	Description of research question and justification for undertaking the trial, including summary of relevant studies (published and unpublished) examining benefits and harms for each intervention	4-5
Background and rationale: choice of comparators	#6b	Explanation for choice of comparators	7
Objectives	#7	Specific objectives or hypotheses	4-5
Trial design	#8	Description of trial design including type of trial (eg, parallel group, crossover, factorial, single group), allocation ratio, and framework (eg, superiority, equivalence, non-inferiority, exploratory)	4

For peer review only

**Methods: Participants, interventions, and outcomes**			
Study setting	#9	Description of study settings (eg, community clinic, academic hospital) and list of countries where data will be collected. Reference to where list of study sites can be obtained	6, supplementary data, NCT03970356
Eligibility criteria	#10	Inclusion and exclusion criteria for participants. If applicable, eligibility criteria for study centres and individuals who will perform the interventions (eg, surgeons, psychotherapists)	6
Interventions: description	#11a	Interventions for each group with sufficient detail to allow replication, including how and when they will be administered	7-8
Interventions: modifications	#11b	Criteria for discontinuing or modifying allocated interventions for a given trial participant (eg, drug dose change in response to harms, participant request, or improving / worsening disease)	n/a
Interventions: adherance	#11c	Strategies to improve adherence to intervention protocols, and any procedures for monitoring adherence (eg, drug tablet return; laboratory tests)	n/a
Interventions: concomitant care	#11d	Relevant concomitant care and interventions that are permitted or prohibited during the trial	n/a
Outcomes	#12	Primary, secondary, and other outcomes, including the specific measurement variable (eg, systolic blood pressure), analysis metric (eg, change from baseline, final value, time to event), method of aggregation (eg, median, proportion), and time point for each outcome. Explanation of the clinical relevance of chosen efficacy and harm outcomes is strongly recommended	8
Participant timeline	#13	Time schedule of enrolment, interventions (including any run-ins and washouts),	Figure 3

For peer review only

		assessments, and visits for participants. A schematic diagram is highly recommended (see Figure)	
Sample size	#14	Estimated number of participants needed to achieve study objectives and how it was determined, including clinical and statistical assumptions supporting any sample size calculations	6-7
Recruitment	#15	Strategies for achieving adequate participant enrolment to reach target sample size	6
**Methods: Assignment of interventions (for controlled trials)**			
Allocation: sequence generation	#16a	Method of generating the allocation sequence (eg, computer-generated random numbers), and list of any factors for stratification. To reduce predictability of a random sequence, details of any planned restriction (eg, blocking) should be provided in a separate document that is unavailable to those who enrol participants or assign interventions	7
Allocation concealment mechanism	#16b	Mechanism of implementing the allocation sequence (eg, central telephone; sequentially numbered, opaque, sealed envelopes), describing any steps to conceal the sequence until interventions are assigned	n/a
Allocation: implementation	#16c	Who will generate the allocation sequence, who will enrol participants, and who will assign participants to interventions	n/a
Blinding (masking)	#17a	Who will be blinded after assignment to interventions (eg, trial participants, care providers, outcome assessors, data analysts), and how	n/a

For peer review only

Blinding (masking): emergency unblinding	#17b	If blinded, circumstances under which unblinding is permissible, and procedure for revealing a participant's allocated intervention during the trial	n/a
**Methods: Data collection, management, and analysis**			
Data collection plan	#18a	Plans for assessment and collection of outcome, baseline, and other trial data, including any related processes to promote data quality (eg, duplicate measurements, training of assessors) and a description of study instruments (eg, questionnaires, laboratory tests) along with their reliability and validity, if known. Reference to where data collection forms can be found, if not in the protocol	8-9
Data collection plan: retention	#18b	Plans to promote participant retention and complete follow-up, including list of any outcome data to be collected for participants who discontinue or deviate from intervention protocols	n/a
Data management	#19	Plans for data entry, coding, security, and storage, including any related processes to promote data quality (eg, double data entry; range checks for data values). Reference to where details of data management procedures can be found, if not in the protocol	9
Statistics: outcomes	#20a	Statistical methods for analysing primary and secondary outcomes. Reference to where other details of the statistical analysis plan can be found, if not in the protocol	9
Statistics: additional analyses	#20b	Methods for any additional analyses (eg, subgroup and adjusted analyses)	9
Statistics: analysis population and missing data	#20c	Definition of analysis population relating to protocol non-adherence (eg, as randomised	9

For peer review only

		analysis), and any statistical methods to handle missing data (eg, multiple imputation)	
**Methods: Monitoring**			
Data monitoring: formal committee	#21a	Composition of data monitoring committee (DMC); summary of its role and reporting structure; statement of whether it is independent from the sponsor and competing interests; and reference to where further details about its charter can be found, if not in the protocol. Alternatively, an explanation of why a DMC is not needed	11
Data monitoring: interim analysis	#21b	Description of any interim analyses and stopping guidelines, including who will have access to these interim results and make the final decision to terminate the trial	11
Harms	#22	Plans for collecting, assessing, reporting, and managing solicited and spontaneously reported adverse events and other unintended effects of trial interventions or trial conduct	11
Auditing	#23	Frequency and procedures for auditing trial conduct, if any, and whether the process will be independent from investigators and the sponsor	n/a
**Ethics and dissemination**			
Research ethics approval	#24	Plans for seeking research ethics committee / institutional review board (REC / IRB) approval	11
Protocol amendments	#25	Plans for communicating important protocol modifications (eg, changes to eligibility criteria, outcomes, analyses) to relevant parties (eg, investigators, REC / IRBs, trial participants, trial registries, journals, regulators)	11
Consent or assent	#26a	Who will obtain informed consent or assent from potential trial participants or authorised surrogates, and how (see Item 32)	6

For peer review only

Consent or assent: ancillary studies	#26b	Additional consent provisions for collection and use of participant data and biological specimens in ancillary studies, if applicable	n/a
Confidentiality	#27	How personal information about potential and enrolled participants will be collected, shared, and maintained in order to protect confidentiality before, during, and after the trial	8-9
Declaration of interests	#28	Financial and other competing interests for principal investigators for the overall trial and each study site	12
Data access	#29	Statement of who will have access to the final trial dataset, and disclosure of contractual agreements that limit such access for investigators	n/a
Ancillary and post trial care	#30	Provisions, if any, for ancillary and post-trial care, and for compensation to those who suffer harm from trial participation	n/a
Dissemination policy: trial results	#31a	Plans for investigators and sponsor to communicate trial results to participants, healthcare professionals, the public, and other relevant groups (eg, via publication, reporting in results databases, or other data sharing arrangements), including any publication restrictions	11
Dissemination policy: authorship	#31b	Authorship eligibility guidelines and any intended use of professional writers	n/a
Dissemination policy: reproducible research	#31c	Plans, if any, for granting public access to the full protocol, participant-level dataset, and statistical code	n/a

Appendices

Informed consent materials	#32	Model consent form and other related documentation given to participants and authorised surrogates	n/a

For peer review only

Biological specimens	#33	Plans for collection, laboratory evaluation, and storage of biological specimens for genetic or molecular analysis in the current trial and for future use in ancillary studies, if applicable	n/a

None The SPIRIT Explanation and Elaboration paper is distributed under the terms of the Creative Commons Attribution License CC-BY-NC. This checklist can be completed online using https://www.goodreports.org/, a tool made by the EQUATOR Network in collaboration with Penelope.ai

For peer review only

BMJ Open

A multifaceted antibiotic stewardship intervention using a participatory-action-research approach to improve antibiotic prescribing for urinary tract infections in frail elderly (ImpresU): study protocol for a European qualitative study followed by a pragmatic cluster randomised controlled trial.

Journal:	*BMJ Open*
Manuscript ID	bmjopen-2021-052552.R1
Article Type:	Protocol
Date Submitted by the Author:	07-Sep-2021

Complete List of Authors:	Hartman, Esther; Amsterdam UMC Locatie VUmc, Medicine for older people, Amsterdam Public Health Research Institute; Utrecht University, Julius Center for Health Sciences and Primary Care, University Medical Center Utrecht<br>Groen, Wim; Amsterdam UMC Locatie VUmc, Medicine for older people, Amsterdam Public Health Research Institute<br>Heltveit-Olsen, Silje ; University of Oslo, The Antibiotic Centre for Primary Care, Department of General Practice, Institute of Health and Society<br>Lindbaek, Morten; University of Oslo, The Antibiotic Centre for Primary Care, Department of General Practice, Institute of Health and Society<br>Hoye, Sigurd; University of Oslo, The Antibiotic Centre for Primary Care, Department of General Practice, Institute of Health and Society<br>Sundvall, Pär-Daniel ; University of Gothenburg, General Practice/Family Medicine, School of Public Health and Community Medicine, Institute of Medicine, Sahlgrenska Academy; Region Västra Götaland, Research, Education, Development & Innovation, Primary Health Care<br>Gunnarsson, Ronny; University of Gothenburg, General Practice/Family Medicine, School of Public Health and Community Medicine, Institute of Medicine, Sahlgrenska Academy; Region Västra Götaland, Research, Education, Development & Innovation, Primary Health Care<br>Skoglund, Ingmarie; University of Gothenburg, General Practice/Family Medicine, School of Public Health and Community Medicine, Institute of Medicine, Sahlgrenska Academy; Region Västra Götaland, Research, Education, Development & Innovation, Primary Health Care<br>Snaebjörnsson Arnljots, Egill ; University of Gothenburg, General Practice/Family Medicine, School of Public Health and Community Medicine, Institute of Medicine, Sahlgrenska Academy; Region Västra Götaland, Research, Education, Development & Innovation, Primary Health Care<br>Godycki-Cwirko , Maciej; Medical University of Lodz, Centre for Family and Community Medicine, the Faculty of Health Sciences<br>Kowalczyk, Anna; Medical University of Lodz, Centre for Family and Community Medicine, the Faculty of Health Sciences<br>Platteel, Tamara ; Utrecht University, Julius Center for Health Sciences

	and Primary Care, University Medical Center Utrecht<br>Zuithoff, Nicolaas; Utrecht University, Julius Center for Health Sciences and Primary Care, University Medical Center Utrecht<br>Monnier, Annelie; Amsterdam UMC Locatie VUmc, Medicine for older people, Amsterdam Public Health Research Institute<br>Verheij, Theo; Utrecht University, Julius Center for Health Sciences and Primary Care, University Medical Center Utrecht<br>Hertogh, Cees; Amsterdam UMC Locatie VUmc, Medicine for older people, Amsterdam Public Health Research Institute<br>van de Pol, Alma; Utrecht University, Julius Center for Health Sciences and Primary Care, University Medical Center Utrecht
**Primary Subject Heading**:	General practice / Family practice
Secondary Subject Heading:	Geriatric medicine, Infectious diseases
Keywords:	Urinary tract infections < UROLOGY, PRIMARY CARE, MEDICAL EDUCATION & TRAINING

For peer review only - http://bmjopen.bmj.com/site/about/guidelines.xhtml

I, the Submitting Author has the right to grant and does grant on behalf of all authors of the Work (as defined in the below author licence), an exclusive licence and/or a non-exclusive licence for contributions from authors who are: i) UK Crown employees; ii) where BMJ has agreed a CC-BY licence shall apply, and/or iii) in accordance with the terms applicable for US Federal Government officers or employees acting as part of their official duties; on a worldwide, perpetual, irrevocable, royalty-free basis to BMJ Publishing Group Ltd ("BMJ") its licensees and where the relevant Journal is co-owned by BMJ to the co-owners of the Journal, to publish the Work in this journal and any other BMJ products and to exploit all rights, as set out in our licence.

The Submitting Author accepts and understands that any supply made under these terms is made by BMJ to the Submitting Author unless you are acting as an employee on behalf of your employer or a postgraduate student of an affiliated institution which is paying any applicable article publishing charge ("APC") for Open Access articles. Where the Submitting Author wishes to make the Work available on an Open Access basis (and intends to pay the relevant APC), the terms of reuse of such Open Access shall be governed by a Creative Commons licence – details of these licences and which Creative Commons licence will apply to this Work are set out in our licence referred to above.

Other than as permitted in any relevant BMJ Author's Self Archiving Policies, I confirm this Work has not been accepted for publication elsewhere, is not being considered for publication elsewhere and does not duplicate material already published. I confirm all authors consent to publication of this Work and authorise the granting of this licence.

For peer review only

TITLE

A multifaceted antibiotic stewardship intervention using a participatory-action-research approach to improve antibiotic prescribing for urinary tract infections in frail elderly (ImpresU): study protocol for a European qualitative study followed by a pragmatic cluster randomised controlled trial.

AUTHORS

Esther A.R. Hartman[1,2], Wim G. Groen[1,], Silje Rebekka Heltveit-Olsen[3], Morten Lindbæk[3], Sigurd Høye[3], Pär-Daniel Sundvall[4, 5], Ronny Gunnarsson[4, 5], Ingmarie Skoglund[4, 5] , Egill Snaebjörnsson Arnljots[4, 5], Maciej Godycki-Cwirko[6], Anna Kowalczyk[6], Tamara N. Platteel[2] , Nicolaas P.A. Zuithoff[2], Annelie A. Monnier[1], Theo J.M. Verheij[2], Cees M.P.M. Hertogh[1] Alma C. van de Pol[2]

Corresponding author: Esther Hartman, Department of medicine for older people, Amsterdam UMC location VUmc, Room B-353, Van der Boechorstraat 7, 1081 BT, Amsterdam, e.hartman1@amsterdamumc.nl

Affiliations

[1] Department of medicine for older people, Amsterdam Public Health Research Institute, Amsterdam UMC, Vrije Universiteit Amsterdam, the Netherlands
[2] Julius Center for Health Sciences and Primary Care, University Medical Center Utrecht, Utrecht University, the Netherlands.
[3] The Antibiotic Centre for Primary Care, Department of General Practice, Institute of Health and Society, University of Oslo, Norway
[4]General Practice/Family Medicine, School of Public Health and Community Medicine, Institute of Medicine, Sahlgrenska Academy, University of Gothenburg, Gothenburg, Sweden
[5]Research, Education, Development & Innovation, Primary Health Care, Region Västra Götaland, Sweden
[6] Centre for Family and Community Medicine, the Faculty of Health Sciences, the Medical University of Lodz, Poland

Word count: 3515

For peer review only - http://bmjopen.bmj.com/site/about/guidelines.xhtml

ABSTRACT

Introduction Almost 60% of antibiotics in frail elderly are prescribed for alleged urinary tract infections (UTIs). A substantial part of this comprises prescriptions in case of non-specific symptoms or asymptomatic bacteriuria, for which the latest guidelines promote restrictiveness with antibiotics. We aim to reduce inappropriate antibiotic use for UTIs through an antibiotic stewardship intervention (ASI) that encourages to prescribe according to these guidelines. To develop an effective ASI, we first need a better understanding of the complex decision-making process concerning suspected UTIs in frail elderly. Moreover, the implementation approach requires tailoring to the heterogeneous elderly care setting.

Methods and analysis First, we conduct a qualitative study to explore factors contributing to antibiotic prescribing for UTIs in frail elderly, using semi-structured interviews with general practitioners, nursing staff, patients, and informal caregivers. Next, we perform a pragmatic cluster randomised controlled trial in elderly care organisations. A multifaceted ASI is implemented in the intervention group; the control group receives care as usual. The ASI is centred around a decision tool that promotes restrictive antibiotic use, supported by a toolbox with educational materials. For the implementation, we use a modified participatory-action-research approach, guided by the results of the qualitative study. The primary outcome is the number of antibiotic prescriptions for suspected UTIs. We aim to recruit 34 clusters with in total 680 frail elderly residents ≥ 70 years. Data collection takes place during a 5-month baseline period and a 7-month follow-up period. Finally, we perform a process evaluation. The study has been delayed for 6 months due to COVID-19 and is expected to end in July 2021.

Ethics and dissemination Ethical approvals and/or waivers were obtained from the ethical committees in Poland, the Netherlands, Norway, and Sweden. The results will be disseminated through publication in peer-reviewed journals and conference presentations.

Trial Registration number NCT03970356

KEYWORDS

Urinary tract infections; Primary care; Medical education & Training

For peer review only

For peer review only - http://bmjopen.bmj.com/site/about/guidelines.xhtml

ARTICLE SUMMARY

Strengths and limitations of this study

- The qualitative study allows for a comprehensive analysis of the factors at play in decision-making on UTIs in frail elderly, which is essential to make progress in antibiotic stewardship in this setting.
- The pragmatic approach with its diverse international setting offers both broad applicability of results in general practice- and elderly care medicine, and gives a chance to evaluate country-specific outcomes.
- The use of participatory action research (PAR) embedded within a cluster randomised trial is infrequent, and may offer valuable insights for future trials; however, a limitation of the tailored approach is that the results will not be exactly replicable.
- The process evaluation of the PAR approach will provide guidance for implementation in daily practice, including a toolbox with supportive educational materials.
- The COVID-19 pandemic began in the midst of the implementation process, undoubtedly affecting the process and results.

For peer review only

For peer review only - http://bmjopen.bmj.com/site/about/guidelines.xhtml

INTRODUCTION

Background and rationale

Suspected urinary tract infections (UTIs) account for the majority of antibiotic prescriptions in frail elderly. In recent years, consensus has been reached that non-specific symptoms in frail elderly are often not attributable to UTIs and do not require an antibiotic prescription.[1, 2] However, it is estimated that between 32 and 62% of prescriptions for UTIs are inappropriately given to patients with only non-specific symptoms.[3, 4](Sundvall, NAPCRG conference 2017, unpublished) International efforts have been made to improve appropriate antibiotic prescribing: a decision tool to support physician's prescribing decisions was developed,[1] and recent guidelines promote restrictive antibiotic use for UTIs in frail elderly.[2] However, international evidence from a randomised controlled trial on their efficacy in reducing inappropriate antibiotic use for UTIs is currently lacking.

Antibiotic prescribing decisions are known to be complex and influenced by many social and organisational factors.[5, 6] In UTIs in frail elderly, this is further complicated by diagnostic uncertainties. First frail elderly patients often present with non-specific symptoms. These symptoms should be evaluated for other causes but are often directly attributed to UTIs.[1, 2, 4, 7, 8] Second, interpretation of urinalysis is clouded by the high prevalence of asymptomatic bacteriuria, for which antibiotics are not needed.[2, 7] A rigorous behavioural change is required from multiple health care professionals to improve antibiotic prescribing in this population. In order to develop effective antibiotic stewardship interventions (ASIs), it is essential to better understand the complex process leading to the decision to (not) prescribe antibiotics for alleged UTIs. Given the large variety in the organisation of elderly care, it is unlikely that a uniform ASI is effective.[9] Participatory action research (PAR) is a promising method that actively involves the health care professionals to implement an ASI tailored to their setting, while accounting for local barriers and facilitators.[10]

We set out to evaluate whether a multifaceted ASI is effective in reducing antibiotic prescribing for UTIs in frail elderly in various long-term care settings (in Poland, the Netherlands, Norway, and Sweden). To accomplish the substantial behavioural changes that are needed, we believe we need a combination of qualitative methods for exploration and a PAR approach for implementation. First, we perform a qualitative study with semi-structured interviews to develop a conceptual model of factors contributing to antibiotic prescribing decisions in this population. Then we conduct a cluster randomised controlled trial (RCT) in frail elderly in care homes attended by general practitioners (GPs) using PAR to implement an ASI. Finally, we conduct a process evaluation.

Objectives

For peer review only - http://bmjopen.bmj.com/site/about/guidelines.xhtml

- Obtain insights into all relevant factors that contribute to antibiotic prescribing for UTIs in frail elderly.
- Develop a conceptual model integrating these identified factors to guide the development of ASI for UTIs in frail elderly.
- Study the effects of the implementation of a multifaceted ASI on antibiotic prescription rates for UTIs in frail elderly.
- Evaluate the implementation process to understand the cluster RCT outcomes, and the added value of the PAR approach to implement ASIs.

METHODS AND ANALYSIS

The Improving antibiotic Prescribing for Urinary tract infections in frail elderly (ImpresU) study consists of a qualitative study and a cluster RCT. Their integration is shown in figure 1.

Qualitative study

The aims are to explore all relevant factors that contribute to antibiotic (non-)prescribing for UTIs in frail elderly, and to integrate these into a conceptual model to guide the development of effective ASIs.

Design and setting

An exploratory qualitative study using semi-structured interviews is conducted in Poland, the Netherlands, Norway, and Sweden. Interviews are conducted with representatives of three relevant stakeholder groups in the setting of elderly care at home and in institutions: 1) GPs, 2) nursing staff, and 3) patients and informal caregivers.

Eligibility criteria, recruitment and sample size

Recruitment takes place through the networks of the research teams per country. We use purposive sampling to reach variation within the representatives of each stakeholder group (e.g. in setting, years of experience for health care professionals). All participants need to be capable and willing to provide informed consent and communicate personal thoughts in the local language. Patients need to be 70 years or older, and are not recruited during the acute phase of a disease. The aim is to conduct approximately 60 interviews (i.e. 15 per country), preferably equally distributed over the three stakeholder groups.

Data collection and management

Topic lists and interview guides are designed based on literature and (clinical) experience from the researchers.[6] Pilot interviews are performed in each country to verify the appropriateness and completeness of the topic lists. All interviews are conducted in the native language and audio-recorded. Basic demographic data (e.g., gender, age) of

For peer review only

participants are collected. Collected data and transcripts are pseudonymised, using a code for each participant.

Data analysis

Data are analysed with use of the framework method,[11] which consists of the following steps: 1) Interviews are transcribed verbatim and translated into English. 2) The researchers (re)read the interviews for familiarisation. 3) Two researchers independently code a first batch of interviews. 4) Through consensus, a preliminary framework is formed. 5) The remaining interviews are coded using the framework; additions and changes are discussed within the research team. 6) Data are organised in a framework matrix. 7) Data are interpreted, and a conceptual model of factors is derived from the matrix.

<u>Cluster randomised controlled trial</u>

The trial aims to evaluate whether a decision tool for restrictive antibiotic use, implemented using a PAR-approach, reduces antibiotic prescribing for UTIs in frail elderly. For this report, we used the SPIRIT reporting guidelines.[12]

Design and setting

A cluster RCT is performed in nursing homes in Poland, the Netherlands, Norway and Sweden, and in residential care homes and home care organisations in the Netherlands, attended by GPs. More details on the setting are provided in the Data Supplement 1. The cluster and unit of randomisation is the care organisation linked to the GP practice; one care organisation may be attended by multiple GP practices or vice versa. In the final months of the study period, a process evaluation is performed.

Eligibility criteria and recruitment

Recruitment of clusters is performed through the networks of the research groups in Poland, the Netherlands, Norway and Sweden. The care organisations identify eligible patients, provide written study information, and ask whether they may be approached by the research team. Written informed consent from patients (or representatives in case of legal incapacity) is obtained by a visiting researcher or nurse.

For inclusion, patients need to be 70 years or older, have physical and/or mental disabilities and ADL dependency requiring care, do not use prophylactic antibiotics, do not receive hospice care and are estimated not to have a very limited life expectancy (≤1 month). Patients are excluded when they start prophylactic antibiotics, start receiving hospice care, have a limited life expectancy (≤1 month), pass away, or move away from the cluster. Patients need to be included for at least two months to contribute data to the study.

Sample size

For peer review only - http://bmjopen.bmj.com/site/about/guidelines.xhtml

The baseline incidence of UTI prescriptions is assumed to be 0.75 per patient-year.[13-16] It has been shown that between 32% and 62% of these prescriptions are inappropriate, i.e. not based on specific signs and symptoms.[3, 4](Sundvall NAPCRG conference 2017, unpublished) After implementation of the algorithm, we assume the prescription rate to be reduced from 0.75 to 0.4 prescriptions per person-year. The intracluster correlation coefficient (ICC) is expected to be 0.06, in line with related studies in the primary care and nursing home setting.[17, 18]

For the sample size calculation, a Wilcoxon Test with an adjustment for cluster randomisation was performed. With an expected cluster size of 10 patients, each contributing 7 months in the follow-up period, one-sided testing, alpha of 0.05, and power of 0.8, it is estimated that 333 patients are needed, translating into a minimum of 34 clusters. To compensate for loss to follow-up, we assume 20 patients per cluster are needed. In sum, we aim to include 34 participating clusters, i.e. 9 in each country, with in total 680 patients.

Randomisation and blinding

Clusters are randomised to intervention or usual care, using SAS software v9.4 by an independent data manager.[19] Block randomisation is used to assign clusters to intervention or control in each country, stratified on cluster size (small/medium/large). Due to the nature of the intervention, blinding is not possible; however, the aims of the study outcomes are not explicitly stated to the control clusters to avoid contamination.

Intervention

The intervention clusters receive a multifaceted ASI. The control clusters provide care as usual. The intervention period was intended to last 4 months. After a month, it was interrupted by the first wave of the COVID-19 pandemic, resulting in a 6-month pause. Upon restart in September 2020, the pragmatic choice was made to restart the intervention period with a duration of 2-3 months, depending on the local situation.

Decision tool & Toolbox

At the core of the ASI is a decision tool to guide the use of antibiotics for suspected UTIs in frail elderly (Data Supplement 2).[1] It promotes an active monitoring approach in case only nonspecific symptoms are present. This decision tool is incorporated in the Dutch UTI guideline for elderly care medicine and congruent with the Swedish and Norwegian UTI guidelines.[20-22] To support the implementation of the decision tool, a toolbox of educational materials is composed (Figure 2 and Data Supplement 3). First a generic toolbox is designed, centred around the decision tool. Next, it is tailored to become country-specific by the local researchers, based on the qualitative study data and any locally available materials. During the intervention period, further tailoring may take place within the participating cluster itself (Figure 2).

For peer review only - http://bmjopen.bmj.com/site/about/guidelines.xhtml

Implementation: modified PAR approach

The intervention is tailored based on an analysis of the interview data to identify country-specific barriers and facilitators. For example, the roles of the health care professionals and knowledge gaps in care for UTIs differ per country and need to be targeted accordingly. During the intervention period, the researchers and health care professionals together go through a cyclical process of reflection, planning and action during sessions for education and evaluation. These sessions combine a top-down and bottom-up approach; both education on the decision tool and any knowledge gaps identified in the qualitative study, as well as reflection and planning for local implementation. The aim is to go through at least two PAR cycles in each cluster, and to actively involve physicians as well as nursing staff. Further tailoring may be performed in each country and cluster locally.

Outcome assessments

Primary outcome measure:

1. Number of prescriptions of antibiotics for suspected UTIs

Secondary outcome measures:

2. Number of prescriptions of antibiotics for suspected UTIs in office hours
3. Number of incorrect prescriptions of antibiotics for suspected UTIs
4. Incidence of suspected UTIs
5. Incidence of complications within 21 days after each UTI suspicion (presence yes/no of a complication: delirium, pyelonephritis, sepsis and renal failure)
6. Incidence of referral to a hospital within 21 days after each UTI suspicion
7. Incidence of hospital admission within 21 days after each UTI suspicion
8. Mortality
9. Mortality within 21 days after each UTI suspicion

All outcomes are assessed during the follow-up period, and expressed per patient-year.

Data collection

Data are collected during a 5-month baseline period and a 7-month follow-up period, through case report forms (CRFs) completed by the GP, nurse or researcher based on contact with a health care professional or medical file. The timeline for participating clusters and participants is displayed in figure 3.

For each participant, a CRF with patient characteristics is filled in at study entry consisting of items concerning demographics, ADL-dependency measured through the Katz Index of Independence in Activities of Daily Living,[23] and relevant medical history. The GPs prospectively register each UTI suspicion on a short registration form, describing symptoms, diagnostics, and antibiotic treatment (primary and secondary outcomes). After 7 and 21 days, follow-up forms are filled in to assess the course of disease, any change in antibiotic

For peer review only - http://bmjopen.bmj.com/site/about/guidelines.xhtml

treatment, complications, and mortality (primary and secondary outcomes). Overall mortality (secondary outcome) is registered upon exclusion of a patient. Any missing data are retrospectively registered through consultation of GPs, nurses and/or access of the medical records.

Furthermore, anonymised data concerning COVID-19 incidence in the participating care organisations are registered during the follow-up period.

Data management

Data are collected pseudonymised on paper forms, using a study code for each patient. Afterwards, they are electronically registered in the secured online database Research Online, according to ICH-GCP regulations. Research Online has multiple validation rules built into the eCRFs. The data cleaning process is supported by automatically and manually generated queries. At the end of the study, all data will be locked. Dedicated data sets are provided to the researchers for analysis. Data are kept securely for at least 15 years.

Data analysis

The analysis will follow the intention-to-treat principle. For the primary outcome, a generalised linear mixed model for Poisson distributions will be used. In case the assumptions for Poisson distributions are insufficiently met, other distributions will be considered (i.e. negative binomial, generalised Poisson, zero-inflated Poisson). A random intercept will be included to correct for clustering within care facility and/or GP, and an additional random intercept will be included to correct for repeated measurements in patients. When results indicate no or very low clustering at the facility/GP or patient level, the corresponding random intercept will be excluded from the analysis. The comparison between intervention and control group, estimated with the time by treatment interaction, will be reported as Rate Ratio's with a 95% CI and a corresponding p-value. In a second model, pre-specified prognostic factors will be added: age, gender, ADL-dependency, presence of an indwelling catheter, dementia, recurrent UTIs, diabetes mellitus, and kidney disorders. In case there are missing values on baseline variables that were selected as potential confounders, multiple imputation will be considered. Furthermore, subgroup analysis will be performed to assess outcomes in groups per country, with different gender, age, presence of dementia, urinary incontinence, and indwelling catheter.

Process evaluation

A process evaluation is conducted in the care organisations participating in the cluster RCT. The framework described by Saunders et al. is used.[24] Elements that are assessed include fidelity, dose delivered/received, reach, recruitment, and context (including COVID-19 impact). Data are collected through documentation of the intervention process by the researchers, and through questionnaires with closed- and open-ended questions to participating health care personnel. Quantitative data will be reported using descriptive statistics; thematic analysis will be performed on the qualitative data.

For peer review only - http://bmjopen.bmj.com/site/about/guidelines.xhtml

Patient and public involvement

In the qualitative study, patients and informal caregivers are interviewed. These data were taken into account in the intervention implementation in the cluster RCT. In the process of the design of the cluster RCT, a meeting was held with representatives of Network Utrecht, care for the elderly (NUZO), Julius Centre, University Medical Centre Utrecht, the Netherlands. Their suggestions on the protocol were taken into account; for example, on patient-directed toolbox materials.

DISCUSSION

We perform a European qualitative study exploring factors influencing decision making on UTIs in frail elderly, and a pragmatic cluster RCT to assess the effect of a decision tool to improve antibiotic prescribing for UTIs in frail elderly, implemented using a PAR-approach. We believe this combination of methodologies is essential to address the complexity of decision making on UTIs in this population. Drawing lessons from the IMPACT study,[25] we are the first to apply this in a diverse international setting.

The PAR approach for implementation allows us to embrace the heterogeneity of the elderly care settings within and between countries.[26]. With large-scale nursing homes in some countries and small-scale living facilities in others, an identical ASI for each health care professional will not be effective. Tailoring the intervention using PAR promotes bottom-up engagement of health care professionals, thereby enabling the required behavioural changes for lasting effects.

Inherent to the tailored approach are limits in the ability to exactly replicate our results. Nevertheless, the methods are replicable, and we believe our results will be widely applicable. The qualitative study will offer in-depth understanding of the factors involved in decisions on UTI, thereby creating opportunities for future ASI development. Our robust trial design, in line with epidemiological recommendations for evaluating ASI,[27] will provide evidence on the application of the latest UTI guidelines. Furthermore, our process evaluation will generate understanding on the ASI and its components in the various settings, and will provide lessons on the use of PAR in future trials. A practical implementation package will become available, with relevant toolbox materials and lessons for daily practice to be tailored to any setting. A further limitation of our study is that we cannot collect data on overall antibiotic use, as we focus on prospective registration in included patients of suspected UTIs only.

The cluster RCT was interrupted by the first wave of the COVID-19 pandemic during the intervention period, and was forced to pause for 6 months. Restarting required much flexibility from the participating care organisations, where patient care already suffered from the pandemic. Sessions for the intervention meeting had to be repeated (mostly online).

For peer review only - http://bmjopen.bmj.com/site/about/guidelines.xhtml

Furthermore, the 6-month delay and further COVID-19 waves regrettably continue to lead to the passing away of participants, increasing the need for new recruitment. As randomisation takes place per country, we presume effects of COVID-19 on our population characteristics and outcomes, if any, will be balanced between intervention and control clusters.

In conclusion, we aim to evaluate the effectiveness of a multifaceted ASI to reduce antibiotic prescribing for UTIs in frail elderly through a qualitative study and cluster RCT in Poland, the Netherlands, Norway and Sweden. Our tailored approach within the diverse setting is promising to yield broadly applicable results, even if currently challenged by the COVID-19 pandemic.

For peer review only

ETHICS AND DISSEMINATION

Participant safety and monitoring

The cluster RCT is considered low risk, as the intervention corresponds to current guidelines. There is no data monitoring committee, and any SAEs are not reported. No interim analyses are planned. For both the qualitative study and cluster RCT respectively, ethical approval was given by the Committee of Bioethics of the Medical University of Lodz, Poland (RNN/381/18/KE and RNN/260/19/KE), the Regional Committee for Medical and Health Research Ethics in Norway (2018/2191/REK sør-øst A and 2018/2521/REK sør-øst A), and the Swedish Ethical Review Authority (2019-00504 and 2019-00796/1228-18(2019-02541)). In the Netherlands, the Medical Ethics Review Committees established that approval was not required since the Medical Research Involving Human Subjects Act does not apply (2018.500 VU University Medical Centre and WAG/mb/19/012207 University Medical Centre Utrecht). Substantial protocol modifications are communicated to ethical committees and the trial register. Dissemination will take place through publication and presentations. Furthermore, an implementation package will be developed.

Trial Status

Currently, the cluster RCT is ongoing and expected to finish in July 2021. Database lock will take place in September 2021.

Author contributions

CH, TV, ML, PS, MGC conceptualised the study and obtained funding. For the qualitative study, AM drafted the protocol with EH, WG, SHO, ML, SH, PS, IS, ESA, AK, MGC and CH. For the cluster RCT, AP drafted the protocol with EH, WG, SHO, ML, SH, PS, RG, ESA, MGC, AK, TP, NZ, TV, and CH. NZ wrote the statistical analysis plan with EH and AP. EH, WG, AP, AM, TP, SHO, SH, AK, ESA and PS designed the process evaluation. The manuscript was drafted by EH and critically revised by all authors. All authors read and approved the final manuscript.

For peer review only - http://bmjopen.bmj.com/site/about/guidelines.xhtml

Acknowledgements

We wish to thank Sofia Sundvall and Sara Sofia Lithén, research nurses, for their ongoing efforts in data collection. Furthermore, we would like to express our gratitude to the participating general practices and elderly care organisations for their prolonged contributions despite the current pandemic.

Funding statement

This work was supported by JPI AMR with reference number JPIAMR_2017_P007, through national funding agencies: National Science Centre Poland (UMO-2017/25/Z/NZ7/03024), ZonMw the Netherlands(549003002), the Research Council of Norway (284253/H10), and The Swedish Research Council (2017-05975). The Healthcare Board, Region Västra Götaland (N/A) partially funded the Swedish part of the study. The funders have no role in or authority on study design, data collection, management, analysis or interpretation, writing and submission of reports for publication.

Competing interests statement

None declared

FULL REFERENCES

1. van Buul LW, Vreeken HL, Bradley SF, et al. The Development of a Decision Tool for the Empiric Treatment of Suspected Urinary Tract Infection in Frail Older Adults: A Delphi Consensus Procedure. *J Am Med Dir Assoc* 2018;19(9):757-64. doi: 10.1016/j.jamda.2018.05.001 [published Online First: 2018/06/19]
2. Nicolle LE, Gupta K, Bradley SF, et al. Clinical Practice Guideline for the Management of Asymptomatic Bacteriuria: 2019 Update by the Infectious Diseases Society of America. *Clin Infect Dis* 2019;68(10):1611-15. doi: 10.1093/cid/ciz021 [published Online First: 2019/09/12]
3. van Buul LW, Veenhuizen RB, Achterberg WP, et al. Antibiotic prescribing in Dutch nursing homes: how appropriate is it? *J Am Med Dir Assoc* 2015;16(3):229-37. doi: 10.1016/j.jamda.2014.10.003 [published Online First: 2014/12/03]
4. Mayne S, Sundvall PD, Gunnarsson R. Confusion Strongly Associated with Antibiotic Prescribing Due to Suspected Urinary Tract Infections in Nursing Homes. *J Am Geriatr Soc* 2018;66(2):274-81. doi: 10.1111/jgs.15179 [published Online First: 2018/01/11]
5. Colliers A, Coenen S, Remmen R, et al. How do general practitioners and pharmacists experience antibiotic use in out-of-hours primary care? An exploratory qualitative interview study to inform a participatory action research project. *BMJ Open* 2018;8(9):e023154. doi: 10.1136/bmjopen-2018-023154 [published Online First: 2018/10/01]
6. van Buul LW, van der Steen JT, Doncker SM, et al. Factors influencing antibiotic prescribing in long-term care facilities: a qualitative in-depth study. *BMC Geriatr* 2014;14:136. doi: 10.1186/1471-2318-14-136 [published Online First: 2014/12/18]
7. Sundvall PD, Ulleryd P, Gunnarsson RK. Urine culture doubtful in determining etiology of diffuse symptoms among elderly individuals: a cross-sectional study of 32 nursing homes. *BMC Fam Pract* 2011;12:36. doi: 10.1186/1471-2296-12-36 [published Online First: 2011/05/20]
8. Mayne S, Bowden A, Sundvall PD, et al. The scientific evidence for a potential link between confusion and urinary tract infection in the elderly is still confusing - a systematic literature review. *BMC Geriatr* 2019;19(1):32. doi: 10.1186/s12877-019-1049-7 [published Online First: 2019/02/06]

For peer review only - http://bmjopen.bmj.com/site/about/guidelines.xhtml

9. Leykum LK, Pugh JA, Lanham HJ, et al. Implementation research design: integrating participatory action research into randomized controlled trials. *Implement Sci* 2009;4:69. doi: 10.1186/1748-5908-4-69 [published Online First: 2009/10/27]
10. van Buul LW, Sikkens JJ, van Agtmael MA, et al. Participatory action research in antimicrobial stewardship: a novel approach to improving antimicrobial prescribing in hospitals and long-term care facilities. *J Antimicrob Chemother* 2014;69(7):1734-41. doi: 10.1093/jac/dku068 [published Online First: 2014/03/22]
11. Gale NK, Heath G, Cameron E, et al. Using the framework method for the analysis of qualitative data in multi-disciplinary health research. *BMC Med Res Methodol* 2013;13:117. doi: 10.1186/1471-2288-13-117 [published Online First: 2013/09/21]
12. Chan AW, Tetzlaff JM, Gotzsche PC, et al. SPIRIT 2013 explanation and elaboration: guidance for protocols of clinical trials. *BMJ* 2013;346:e7586. doi: 10.1136/bmj.e7586 [published Online First: 2013/01/11]
13. Resultaten van wekelijkse surveillance, referentiecijfers 2011-2015. Surveillance Netwerk Infectieziekten in Verpleeghuizen: Dutch National Institute for Public Health and the Environment.
14. Fagan M, Maehlen M, Lindbaek M, et al. Antibiotic prescribing in nursing homes in an area with low prevalence of antibiotic resistance: compliance with national guidelines. *Scand J Prim Health Care* 2012;30(1):10-5. doi: 10.3109/02813432.2011.629156 [published Online First: 2011/12/23]
15. Ricchizzi E, Latour K, Karki T, et al. Antimicrobial use in European long-term care facilities: results from the third point prevalence survey of healthcare-associated infections and antimicrobial use, 2016 to 2017. *Euro Surveill* 2018;23(46) doi: 10.2807/1560-7917.ES.2018.23.46.1800394 [published Online First: 2018/11/22]
16. Sundvall PD, Stuart B, Davis M, et al. Antibiotic use in the care home setting: a retrospective cohort study analysing routine data. *BMC Geriatr* 2015;15:71. doi: 10.1186/s12877-015-0073-5 [published Online First: 2015/06/26]
17. Campbell MK, Fayers PM, Grimshaw JM. Determinants of the intracluster correlation coefficient in cluster randomized trials: the case of implementation research. *Clin Trials* 2005;2(2):99-107. doi: 10.1191/1740774505cn071oa [published Online First: 2005/11/11]
18. Rutten JJS, van Buul LW, Smalbrugge M, et al. Antibiotic prescribing and non-prescribing in nursing home residents with signs and symptoms ascribed to urinary tract infection (ANNA): study protocol for a cluster randomized controlled trial. *BMC Geriatr* 2020;20(1):341. doi: 10.1186/s12877-020-01662-0 [published Online First: 2020/09/12]
19. SAS Version 9.4 [program]: SAS Institute Inc. SAS and all other SAS Institute Inc. product or service names are registered trademarks or trademarks of SAS Institute Inc., Cary, NC, USA.
20. Läkemedelsbehandling av urinvägsinfektioner i öppenvård - behandlingsrekommendation: Läkemedelsverket, 2017:21-36.
21. Richtlijn Urineweginfecties bij kwetsbare ouderen: Verenso, 2018.
22. Akselsen PEO, S. Urinveisinfeksjoner i sykehjem, versjon 2.2: Antibiotikasenteret for primærmedisin, 2020.
23. Katz S, Ford AB, Moskowitz RW, et al. Studies of Illness in the Aged. The Index of Adl: A Standardized Measure of Biological and Psychosocial Function. *JAMA* 1963;185:914-9. doi: 10.1001/jama.1963.03060120024016 [published Online First: 1963/09/21]
24. Saunders RP, Evans MH, Joshi P. Developing a process-evaluation plan for assessing health promotion program implementation: a how-to guide. *Health Promot Pract* 2005;6(2):134-47. doi: 10.1177/1524839904273387 [published Online First: 2005/04/28]
25. van Buul LW, van der Steen JT, Achterberg WP, et al. Effect of tailored antibiotic stewardship programmes on the appropriateness of antibiotic prescribing in nursing homes. *J Antimicrob Chemother* 2015;70(7):2153-62. doi: 10.1093/jac/dkv051 [published Online First: 2015/03/07]

For peer review only - http://bmjopen.bmj.com/site/about/guidelines.xhtml

26. Van Buul LW, Monnier AA, Sundvall PD, et al. Antibiotic Stewardship in European Nursing Homes: Experiences From the Netherlands, Norway, Poland, and Sweden. *J Am Med Dir Assoc* 2020;21(1):34-40 e1. doi: 10.1016/j.jamda.2019.10.005 [published Online First: 2019/12/04]
27. de Kraker MEA, Abbas M, Huttner B, et al. Good epidemiological practice: a narrative review of appropriate scientific methods to evaluate the impact of antimicrobial stewardship interventions. *Clin Microbiol Infect* 2017;23(11):819-25. doi: 10.1016/j.cmi.2017.05.019 [published Online First: 2017/06/03]

FIGURE LEGENDS

Figure 1: Schematic overview of the interplay between the two studies.
The qualitative study offers insights to tailor the antibiotic stewardship intervention in the cluster randomised controlled trial (RCT), through a country-specific local analysis. The cluster RCT consists of a baseline- and follow-up period for data collection, with an intervention period or usual care in between (the timeline is provided in Figure 3). A process evaluation follows at the end of the cluster RCT.

Figure 2: Toolbox.
The educational materials and targeted stakeholders in the generic toolbox are listed, and the tailoring process is shown.

Figure 3: Timeline of the cluster randomised controlled trial.
The periods of data collection and procedures are shown for the clusters and participating patients.

For peer review only

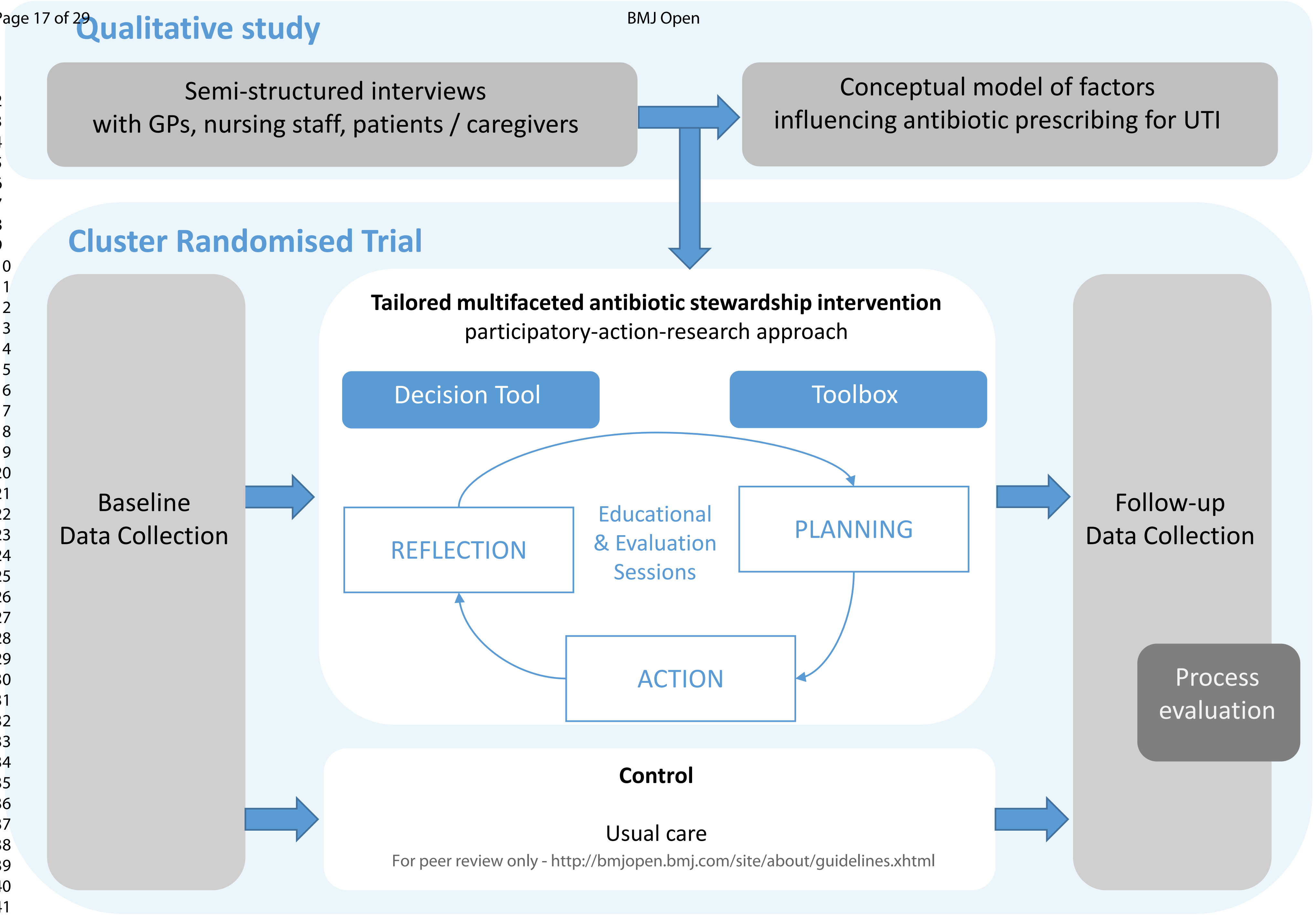

For peer review only - http://bmjopen.bmj.com/site/about/guidelines.xhtml

Materials (targeted stakeholder)	Tailoring process
Information leaflet (GPs, nursing staff, patients)	
Pocket card (GPs, nursing staff)	
Poster (nursing staff, patients)	
Active monitoring checklist (nursing staff)	
Case study (GPs, nursing staff)	
E-learning (nursing staff)	

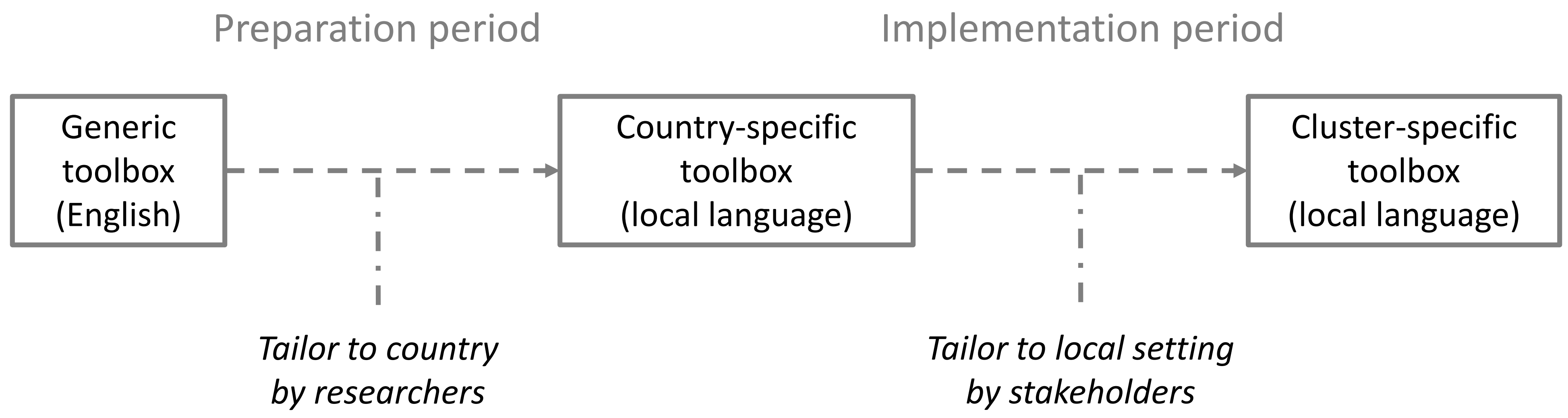

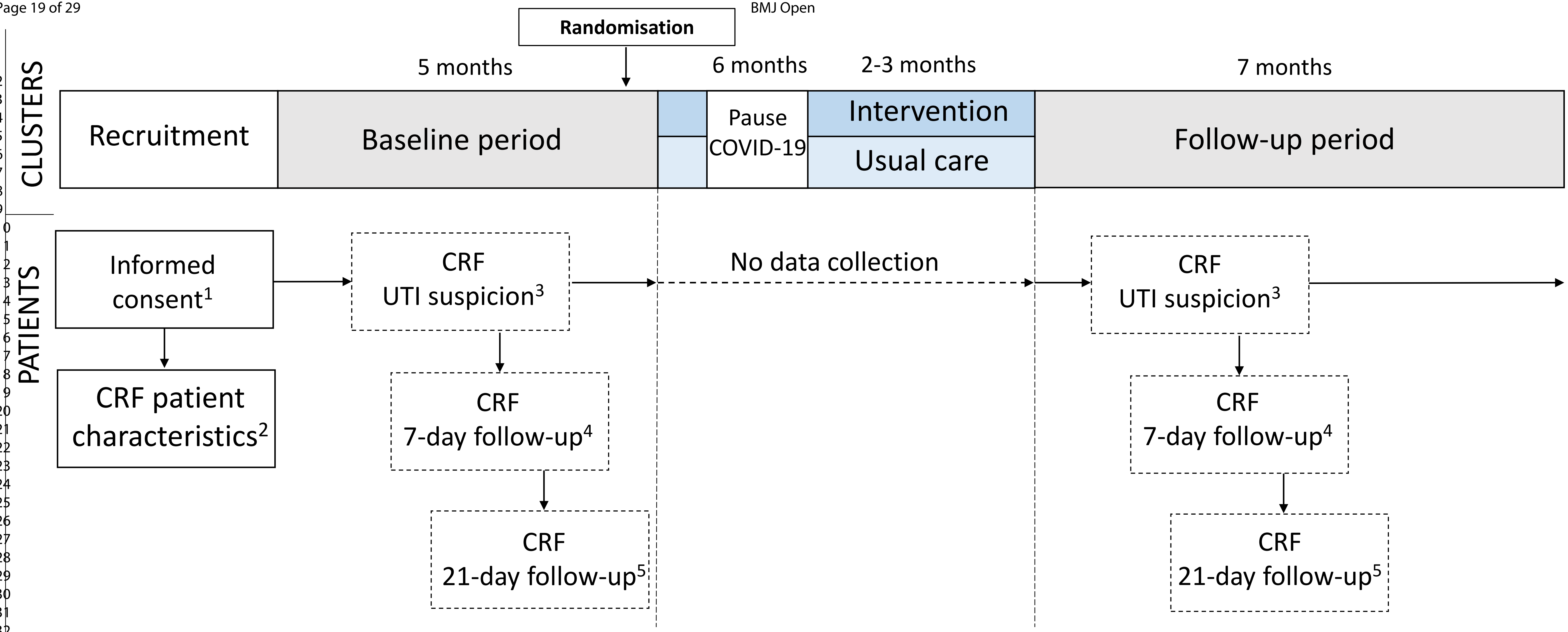

[1] Continuous enrollment throughout the study
[2] Demographics, ADL-dependency, relevant medical history
[3] Symptoms, diagnostics, antibiotic treatment
[4&5] Course of disease and treatment, complications, mortality

For peer review only - http://bmjopen.bmj.com/site/about/guidelines.xhtml

SUPPLEMENTARY DATA

Data supplement 1: Setting of the participating clusters in the cluster RCT

Poland

Participating clusters consist of nursing homes with patients registered to a particular GP primary care centre. Nursing homes in Poland provide living, care, support and educational services to people who require 24-hour care due to their age, illness or disability. Nursing homes may be conducted by local government units, churches, or other associations.

- Nursing care is provided 24-hours a day.
- Patients are registered to a particular GP in a primary care centre.
- Medical services are provided on the general principles of the National Health Fund. Patients can visit their GP in the centre or the GP comes to the nursing home on regular basis and on demand.
- During out-of hours, the regular GP/GP-practice is not be available. Instead, out-of hours service doctors are responsible or an ambulance is called in urgent cases.

The Netherlands

Participating clusters consist of residential care homes or home care organisation and their attending GP practices. This used to be a well-defined GP-attended setting; however, due to recent policy changes the setting is now quite heterogeneous. It does not include nursing homes; specialized elderly care physicians provide medical care in nursing homes.

- Patients receive varying degrees of ADL care, often provided by nurse-assistants with lower educational levels compared to the nursing home setting. Often, nurses are available (on-call). Patients may live in residential care homes or apartment complexes next to it, small-scale living facilities for dementia care, or have “regular” homes with access to home care.
- Medical care is provided by the GP. Often, more than one GP practice is connected to the nursing teams, as patients choose their own GP and their own nursing care organisation. In some residential care homes, the GP visits on a regular basis, for others, the GP is available only on demand.
- During out-of hours, the out-of-hour GP service is available instead of the regular GP.

For peer review only

Norway

Participating clusters consist of nursing homes with nursing home doctors providing medical care. Nursing homes are organised by municipalities, and are reserved for the most vulnerable older persons; those who need 24 hours surveillance and/or are severely dependent in ADL.

- 24-hour care is available at the nursing home from nurses and nurse assistants.
- Medical care is provided by nursing home doctors, with various medical backgrounds, e.g. in general practice or geriatrics.
- During out-of hours, the regular doctor is not available, instead out-of hours service doctors are responsible.

Sweden

Participating clusters consist of nursing homes with medical care provided by GPs. Nursing homes are reserved for the most vulnerable older persons, those who need 24 hours surveillance and/or are severely dependent in ADL.

- Medical care is provided by GPs. Sometimes, more than one GP (practice) is connected to the nursing homes. The GP practices are organised by regional authorities.
- During out-of hours, the regular GP/GP-practice will not be available, instead out-of hours service doctors are responsible.
- Nursing homes are organised by municipalities (separate from the regional authorities). Care is provided by nurse assistants (24-7 service) at the nursing homes. Nurses are available 24-7 but not always present at the nursing homes, as a nurse will be responsible for several nursing homes during evening/nights and weekends.

For peer review only

Data supplement 2: Decision Tool

The decision tool (Van Buul et al. 2018) is the core of the intervention and assists in the decision to prescribe or not prescribe antibiotics. There is a separate tool for patients with and without urinary catheter.

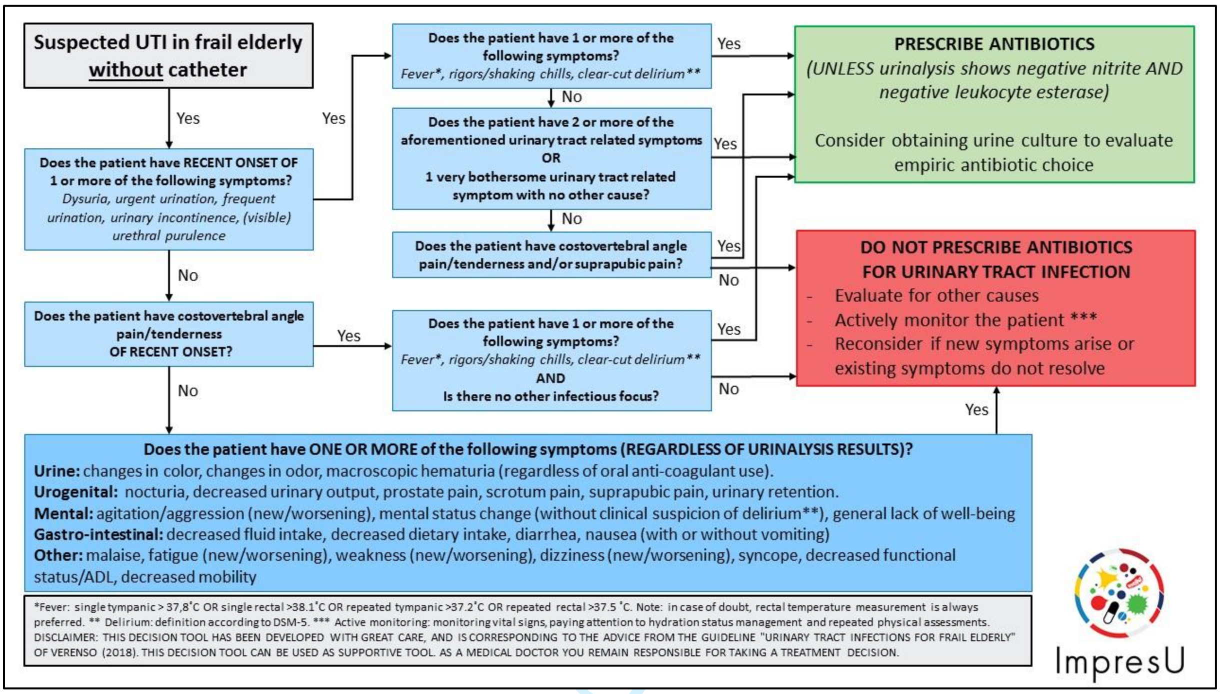

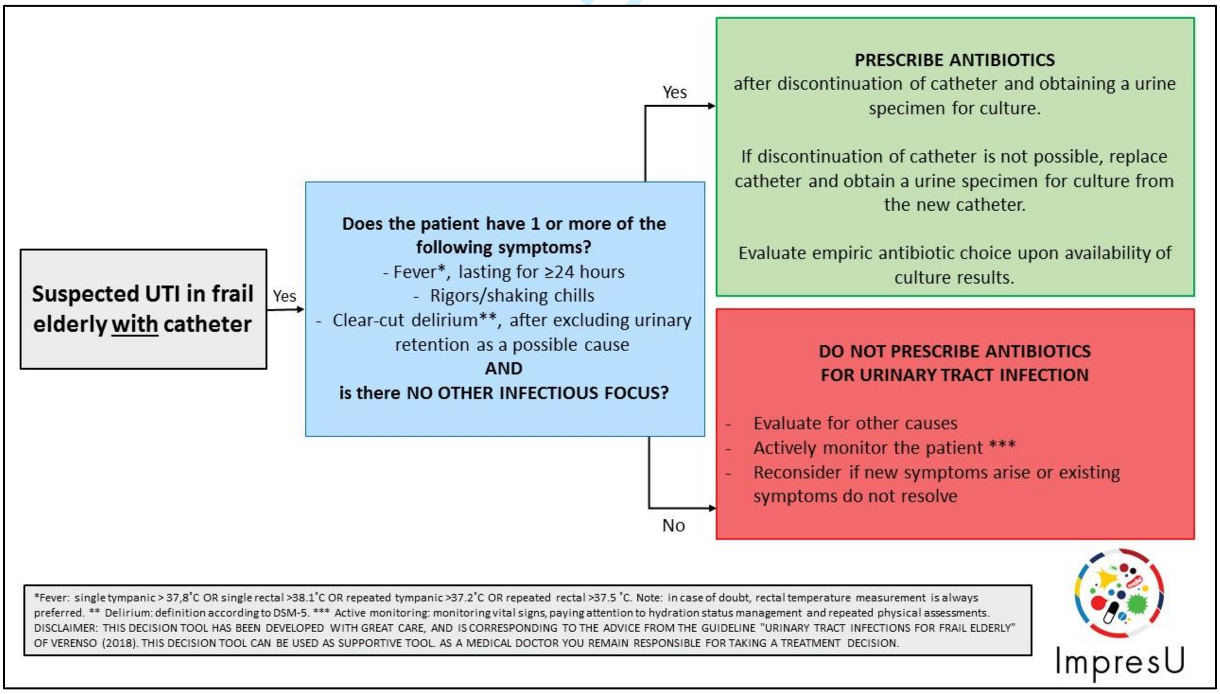

Reference: van Buul LW, Vreeken HL, Bradley SF, et al. The Development of a Decision Tool for the Empiric Treatment of Suspected Urinary Tract Infection in Frail Older Adults: A Delphi Consensus Procedure. J Am Med Dir Assoc 2018;19(9):757-64. doi: 10.1016/j.jamda.2018.05.001 [published Online First: 2018/06/19]

Data supplement 3: Example of toolbox materials

The pocket card for nursing staff is shown. It provides guidance of how to recognize a UTI, when to contact a doctor, and advice for an active monitoring policy. The pocket card is translated for each participating country, and may be tailored to the specific cluster.

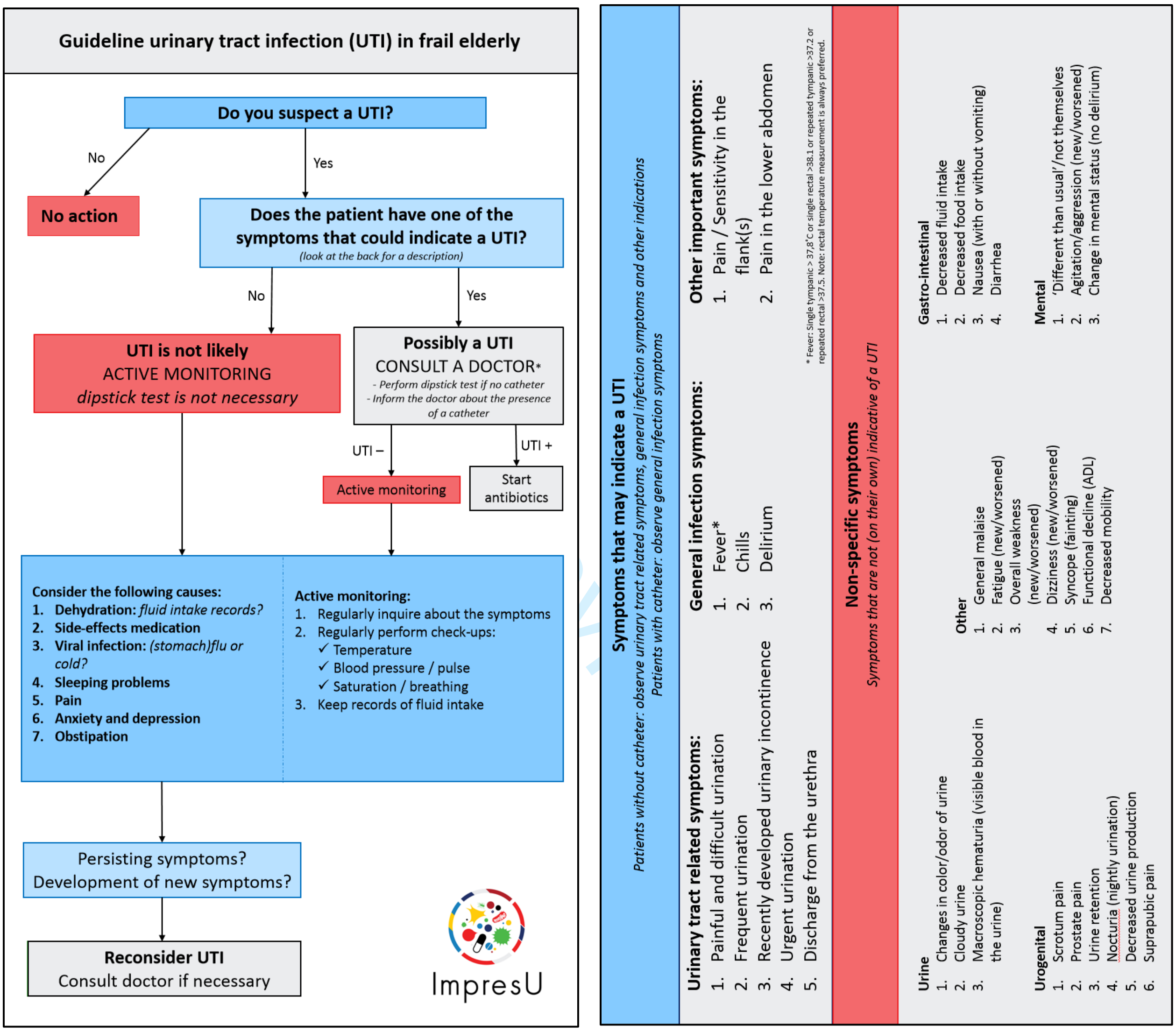

Reporting checklist for protocol of a clinical trial.

Based on the SPIRIT guidelines.

Instructions to authors

Complete this checklist by entering the page numbers from your manuscript where readers will find each of the items listed below.

Your article may not currently address all the items on the checklist. Please modify your text to include the missing information. If you are certain that an item does not apply, please write "n/a" and provide a short explanation.

Upload your completed checklist as an extra file when you submit to a journal.

In your methods section, say that you used the SPIRITreporting guidelines, and cite them as:

Chan A-W, Tetzlaff JM, Gøtzsche PC, Altman DG, Mann H, Berlin J, Dickersin K, Hróbjartsson A, Schulz KF, Parulekar WR, Krleža-Jerić K, Laupacis A, Moher D. SPIRIT 2013 Explanation and Elaboration: Guidance for protocols of clinical trials. BMJ. 2013;346:e7586

		Reporting Item	Page Number
**Administrative information**			
Title	#1	Descriptive title identifying the study design, population, interventions, and, if applicable, trial acronym	1
Trial registration	#2a	Trial identifier and registry name. If not yet registered, name of intended registry	2
Trial registration: data set	#2b	All items from the World Health Organization Trial Registration Data Set	2, NCT03970356
Protocol version	#3	Date and version identifier	V1.9 Dec 10, 2020
Funding	#4	Sources and types of financial, material, and other support	12

For peer review only

Roles and responsibilities: contributorship	#5a	Names, affiliations, and roles of protocol contributors	11
Roles and responsibilities: sponsor contact information	#5b	Name and contact information for the trial sponsor	n/a via corresponding author
Roles and responsibilities: sponsor and funder	#5c	Role of study sponsor and funders, if any, in study design; collection, management, analysis, and interpretation of data; writing of the report; and the decision to submit the report for publication, including whether they will have ultimate authority over any of these activities	12
Roles and responsibilities: committees	#5d	Composition, roles, and responsibilities of the coordinating centre, steering committee, endpoint adjudication committee, data management team, and other individuals or groups overseeing the trial, if applicable (see Item 21a for data monitoring committee)	11 n/a
**Introduction**			
Background and rationale	#6a	Description of research question and justification for undertaking the trial, including summary of relevant studies (published and unpublished) examining benefits and harms for each intervention	4-5
Background and rationale: choice of comparators	#6b	Explanation for choice of comparators	7
Objectives	#7	Specific objectives or hypotheses	4-5
Trial design	#8	Description of trial design including type of trial (eg, parallel group, crossover, factorial, single group), allocation ratio, and framework (eg, superiority, equivalence, non-inferiority, exploratory)	4

For peer review only

**Methods: Participants, interventions, and outcomes**			
Study setting	#9	Description of study settings (eg, community clinic, academic hospital) and list of countries where data will be collected. Reference to where list of study sites can be obtained	6, supplementary data, NCT03970356
Eligibility criteria	#10	Inclusion and exclusion criteria for participants. If applicable, eligibility criteria for study centres and individuals who will perform the interventions (eg, surgeons, psychotherapists)	6
Interventions: description	#11a	Interventions for each group with sufficient detail to allow replication, including how and when they will be administered	7-8
Interventions: modifications	#11b	Criteria for discontinuing or modifying allocated interventions for a given trial participant (eg, drug dose change in response to harms, participant request, or improving / worsening disease)	n/a
Interventions: adherance	#11c	Strategies to improve adherence to intervention protocols, and any procedures for monitoring adherence (eg, drug tablet return; laboratory tests)	n/a
Interventions: concomitant care	#11d	Relevant concomitant care and interventions that are permitted or prohibited during the trial	n/a
Outcomes	#12	Primary, secondary, and other outcomes, including the specific measurement variable (eg, systolic blood pressure), analysis metric (eg, change from baseline, final value, time to event), method of aggregation (eg, median, proportion), and time point for each outcome. Explanation of the clinical relevance of chosen efficacy and harm outcomes is strongly recommended	8
Participant timeline	#13	Time schedule of enrolment, interventions (including any run-ins and washouts),	Figure 3

For peer review only

		assessments, and visits for participants. A schematic diagram is highly recommended (see Figure)	
Sample size	#14	Estimated number of participants needed to achieve study objectives and how it was determined, including clinical and statistical assumptions supporting any sample size calculations	6-7
Recruitment	#15	Strategies for achieving adequate participant enrolment to reach target sample size	6
**Methods: Assignment of interventions (for controlled trials)**			
Allocation: sequence generation	#16a	Method of generating the allocation sequence (eg, computer-generated random numbers), and list of any factors for stratification. To reduce predictability of a random sequence, details of any planned restriction (eg, blocking) should be provided in a separate document that is unavailable to those who enrol participants or assign interventions	7
Allocation concealment mechanism	#16b	Mechanism of implementing the allocation sequence (eg, central telephone; sequentially numbered, opaque, sealed envelopes), describing any steps to conceal the sequence until interventions are assigned	n/a
Allocation: implementation	#16c	Who will generate the allocation sequence, who will enrol participants, and who will assign participants to interventions	n/a
Blinding (masking)	#17a	Who will be blinded after assignment to interventions (eg, trial participants, care providers, outcome assessors, data analysts), and how	n/a

For peer review only

Blinding (masking): emergency unblinding	#17b	If blinded, circumstances under which unblinding is permissible, and procedure for revealing a participant's allocated intervention during the trial	n/a
**Methods: Data collection, management, and analysis**			
Data collection plan	#18a	Plans for assessment and collection of outcome, baseline, and other trial data, including any related processes to promote data quality (eg, duplicate measurements, training of assessors) and a description of study instruments (eg, questionnaires, laboratory tests) along with their reliability and validity, if known. Reference to where data collection forms can be found, if not in the protocol	8-9
Data collection plan: retention	#18b	Plans to promote participant retention and complete follow-up, including list of any outcome data to be collected for participants who discontinue or deviate from intervention protocols	n/a
Data management	#19	Plans for data entry, coding, security, and storage, including any related processes to promote data quality (eg, double data entry; range checks for data values). Reference to where details of data management procedures can be found, if not in the protocol	9
Statistics: outcomes	#20a	Statistical methods for analysing primary and secondary outcomes. Reference to where other details of the statistical analysis plan can be found, if not in the protocol	9
Statistics: additional analyses	#20b	Methods for any additional analyses (eg, subgroup and adjusted analyses)	9
Statistics: analysis population and missing data	#20c	Definition of analysis population relating to protocol non-adherence (eg, as randomised	9

For peer review only

		analysis), and any statistical methods to handle missing data (eg, multiple imputation)	
**Methods: Monitoring**			
Data monitoring: formal committee	#21a	Composition of data monitoring committee (DMC); summary of its role and reporting structure; statement of whether it is independent from the sponsor and competing interests; and reference to where further details about its charter can be found, if not in the protocol. Alternatively, an explanation of why a DMC is not needed	11
Data monitoring: interim analysis	#21b	Description of any interim analyses and stopping guidelines, including who will have access to these interim results and make the final decision to terminate the trial	11
Harms	#22	Plans for collecting, assessing, reporting, and managing solicited and spontaneously reported adverse events and other unintended effects of trial interventions or trial conduct	11
Auditing	#23	Frequency and procedures for auditing trial conduct, if any, and whether the process will be independent from investigators and the sponsor	n/a
**Ethics and dissemination**			
Research ethics approval	#24	Plans for seeking research ethics committee / institutional review board (REC / IRB) approval	11
Protocol amendments	#25	Plans for communicating important protocol modifications (eg, changes to eligibility criteria, outcomes, analyses) to relevant parties (eg, investigators, REC / IRBs, trial participants, trial registries, journals, regulators)	11
Consent or assent	#26a	Who will obtain informed consent or assent from potential trial participants or authorised surrogates, and how (see Item 32)	6

For peer review only

Consent or assent: ancillary studies	#26b	Additional consent provisions for collection and use of participant data and biological specimens in ancillary studies, if applicable	n/a
Confidentiality	#27	How personal information about potential and enrolled participants will be collected, shared, and maintained in order to protect confidentiality before, during, and after the trial	8-9
Declaration of interests	#28	Financial and other competing interests for principal investigators for the overall trial and each study site	12
Data access	#29	Statement of who will have access to the final trial dataset, and disclosure of contractual agreements that limit such access for investigators	n/a
Ancillary and post trial care	#30	Provisions, if any, for ancillary and post-trial care, and for compensation to those who suffer harm from trial participation	n/a
Dissemination policy: trial results	#31a	Plans for investigators and sponsor to communicate trial results to participants, healthcare professionals, the public, and other relevant groups (eg, via publication, reporting in results databases, or other data sharing arrangements), including any publication restrictions	11
Dissemination policy: authorship	#31b	Authorship eligibility guidelines and any intended use of professional writers	n/a
Dissemination policy: reproducible research	#31c	Plans, if any, for granting public access to the full protocol, participant-level dataset, and statistical code	n/a

Appendices

Informed consent materials	#32	Model consent form and other related documentation given to participants and authorised surrogates	n/a

For peer review only

Biological specimens	#33	Plans for collection, laboratory evaluation, and storage of biological specimens for genetic or molecular analysis in the current trial and for future use in ancillary studies, if applicable	n/a

None The SPIRIT Explanation and Elaboration paper is distributed under the terms of the Creative Commons Attribution License CC-BY-NC. This checklist can be completed online using https://www.goodreports.org/, a tool made by the EQUATOR Network in collaboration with Penelope.ai

For peer review only
